# The wider implications of dactylitis beyond psoriatic arthritis

**DOI:** 10.1038/s43856-026-01730-3

**Published:** 2026-07-24

**Authors:** Niccolò Possemato, Marianna Oliva, Alessandra Rai, Nicolò Girolimetto, Francesco Caso, Carlo Salvarani, Antonio Marchesoni, Philip S. Helliwell

**Affiliations:** 1 Department of Rheumatology, Azienda USL-IRCCS Di Reggio Emilia, Reggio Emilia, Italy; 2 Division of Internal Medicine, Azienda Unitaria Sanitaria Locale of Bologna, Bologna, Italy; 3 Department of Clinical Medicine and Surgery, Rheumatology Unit, University of Napoli Federico II, Napoli, Italy; 4 University of Modena and Reggio Emilia, Modena, Italy; 5 Rheumatology, Humanitas San Pio X, Milano, Italy; 6 P.S. Helliwell, MD, PhD, Leeds Institute of Rheumatic and Musculoskeletal Medicine, University of Leeds, Chapel Allerton Hospital, Leeds, UK

**Keywords:** Spondyloarthritis, Rheumatology, Psoriatic arthritis

## Abstract

Dactylitis (from the Greek word *dactylos*, meaning “digit”) is a medical condition commonly known as “sausage digit”, referring to the diffuse swelling of a finger or toe. Dactylitis is most commonly associated with the spectrum of spondyloarthritis, particularly psoriatic arthritis (PsA), and more rarely with other inflammatory conditions such as sarcoidosis and gout. In PsA, dactylitis is associated with increased disease severity and radiographic progression, and its recognition may facilitate early diagnosis and timely treatment. In sarcoid disease, dactylitis is clinically relevant, reflecting granulomatous inflammation and potentially indicating multisystem involvement. Although most commonly linked to inflammatory disorders, it may also manifest in a variety of other conditions, such as trauma, congenital deformities, infectious diseases, and neoplastic processes, all of which can present with a clinically indistinguishable phenotype. This review provides a comprehensive, up-to-date overview of dactylitis, beginning with the origin of the term and its earliest historical and scientific references, and concluding with the latest insights into aetiopathogenesis, classification, anatomy, imaging, and therapeutic strategies. Through the integration of clinical knowledge with new developments in imaging, the paper explores how the study of dactylitis can extend beyond PsA.

## Abstract (web summary)

Possemato, Oliva, et al. review literature on the aetiopathogenesis, anatomy, imaging, and management of dactylitis, a common manifestation of psoriatic arthritis also present with an indistinguishable phenotype in other conditions. They show that it is a multifaceted clinical sign and advances in imaging may enable accurate diagnosis and therapy.

## Introduction

Dactylitis (from the Greek word “*dactylos*”, meaning digit) is a medical condition commonly known as “sausage digit”, referring to the diffuse swelling of a finger or a toe. Nowadays, dactylitis is generally associated with PsA, but the term “sausage digit” can apply to different medical conditions, both inflammatory and non-inflammatory. Conditions unrelated to inflammation, such as trauma, congenital deformities, or neoplastic processes, can result in indistinguishable conditions. It may be associated with infectious diseases, while non-infectious inflammatory causes include gout, sarcoidosis, and spondyloarthritis (SpA) other than PsA. In addition, other conditions can mimic dactylitis, such as sickle cell anemia or pachydermodactyly, a rare, benign form of digital fibromatosis in children or young men.

To fully understand the mechanisms of dactylitis, it is essential to analyze the complex anatomy of the digit. Anatomical dissections and the use of imaging, such as musculoskeletal ultrasound (MSK-US), thanks to the increasing availability of high-frequency probes, have allowed a more precise characterization of these structures and a more confident diagnosis^1^.

### Definition and historical background

The earliest description of dactylitis is attributed to Avicenna in the 11th century, who reported finger swelling in the context of infectious diseases. The classical definition of dactylitis, provided in 1998 by Rothschild BM et al., describes it as a uniform swelling of the digit^2^. Earlier clinical observations also highlighted the contribution of joint and tendon sheath inflammation to the characteristic “sausage-like” appearance^3^. Initially, dactylitis was not considered a clinically distinct or relevant feature of PsA^4^. Its relevance was clearly established with the CASPAR criteria in 2006, where dactylitis (current or past) emerged as a highly specific feature of PsA, observed in 54% of cases^5^.

Dactylitis, however, may be due not only to PsA or other inflammatory conditions, but also to a variety of other disorders, including trauma or congenital deformities^6^. Epidemiological data vary across studies and are influenced by differences in patient populations, with reported prevalence rates of 39% in PsA, 17% in sarcoidosis, 9.5–12% in other SpAs, and 5–9.6% in gout^7^. Furthermore, dactylitis may represent the manifestation of a neoplastic process (particularly osteoid osteoma) or a paraneoplastic syndrome^8,9^.

### Anatomical considerations in dactylitis

In addition to bones and synovial joints, each digit can be identified as having three main regions: the dorsal region, which houses the extensor apparatus; the volar region, which contains two flexor tendons anchored to cortical bone through a pulley system; and the lateral region, consisting of collateral ligaments that contribute to joint stability^10^. Classical anatomical classifications, such as those proposed by Verdan, Kleinert, and Quílez Caballero, allow precise localization of tendon injuries and extensor structures^11,12^.

**Fig. 1:**
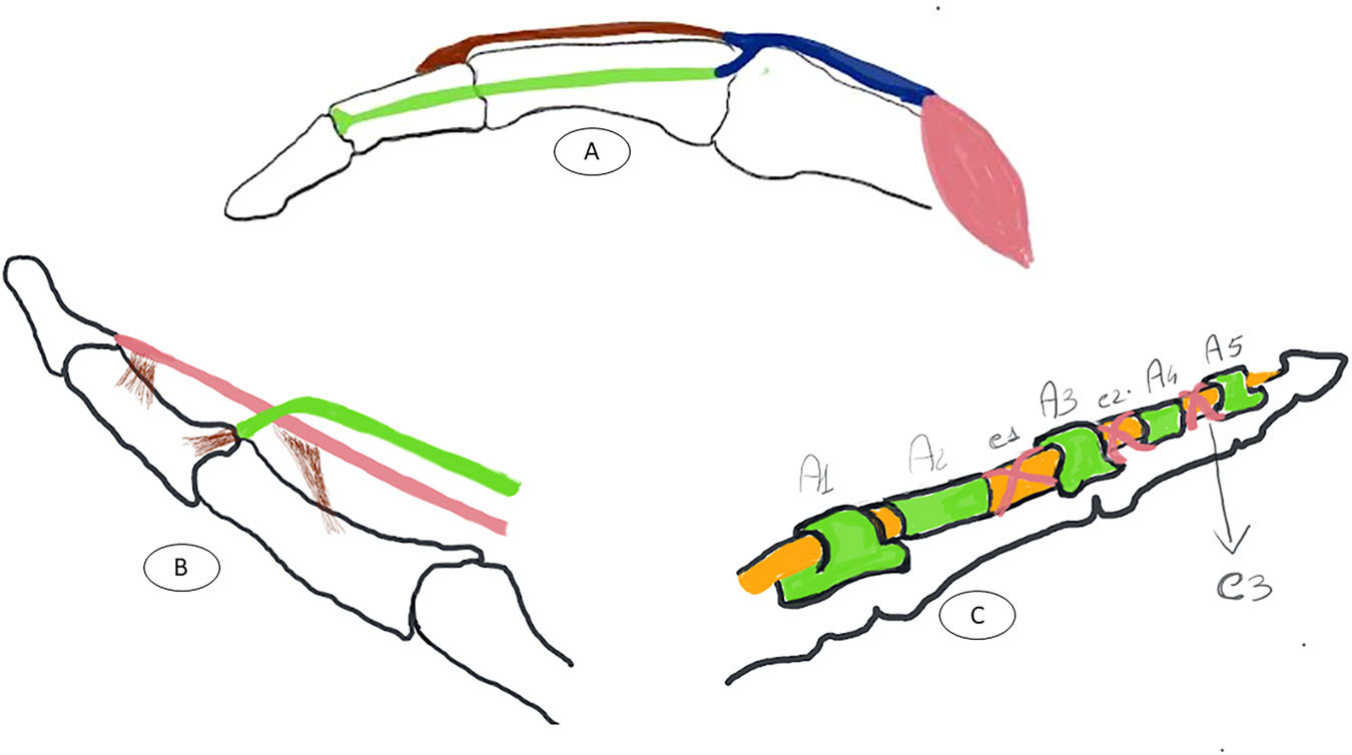
Schematic representation of the digital anatomy relevant to dactylitis, which represent key sites of inflammation in psoriatic arthritis. **A** Extensor apparatus of the finger: central slip (brown), sagittal band of the common extensor tendon (green), extensor tendon (blue), and interosseous muscles (pink) interconnected with the tendinous system. **B** Camper’s chiasm: volar aspect of the finger showing the flexor digitorum profundus (pink), flexor digitorum superficialis (green), and vincula (red) supplying the flexor tendons. **C** Pulley system of the finger: annular pulleys (green), including A1, most commonly associated with trigger finger, and A3, positioned over the volar plate. The cruciate pulleys (pink) are interposed between the annular pulleys, while the flexor tendon system is shown in orange.

A thorough knowledge of digital anatomy is essential, as specific structures represent key targets of inflammation in diseases, such as PsA (Fig. 1).

#### Dorsal region

The extensor apparatus of the finger is a complex functional unit involving the extensor digitorum, lumbrical, and interosseous muscles. A key structure is the extensor hood, which stabilizes the extensor tendon located over the dorsal surface of the proximal phalanx. It includes sagittal bands, oblique and transverse fibers, with sagittal bands playing a major role in maintaining tendon alignment and being frequently involved in inflammatory conditions^10,12^. Distal to the metacarpophalangeal joint, the extensor digitorum communis divides into three parts. The central slip inserts at the base of the middle phalanx, while the lateral bands run along each side of the proximal interphalangeal joint^10^. All bony insertions of the central slip display fibrocartilage. These structures form part of the synovio-entheseal complex, which has been implicated in the pathogenesis of PsA^13,14^

#### Volar region

The volar region of the finger houses two flexor tendons: the flexor digitorum superficialis (FDS) and the flexor digitorum profundus (FDP). These tendons are enclosed within a synovial sheath that extends from the neck of the metacarpal to the distal interphalangeal joint^15^. The fibrous sheath stabilizes tendon position^16^. The intersection of these tendons forms the Camper’s chiasm^12^. The flexor tendons are supplied by the digital arteries through a complex vascular network supported by connective tissue strands, known as vincula^17^. Also located in the volar region, the volar plates are fibrocartilaginous structures that reinforce the stability of the joints. Each finger (except the thumb) typically also contains five annular pulleys (A1–A5) and three cruciform pulleys (C1–C3)^18^. Their function is to prevent tendon bow-stringing during the digit flexion^12,19^.

In PsA, pulleys are relevant sites of inflammation, supporting their role as functional entheses^16^. Notably, in PsA, fibrocartilage of the A2 and A4 pulleys can develop enthesophytes—bony projections at the enthesis—which may represent signs of inflammatory pathology^20^.

#### Lateral structures

Collateral ligaments are the main lateral stabilizers of the digital joints. They are classified as proper collateral ligaments (limiting ulnar and radial deviation) and accessory. They insert into the bone and the volar plate, and chronic microtrauma or inflammation can lead to their structural damage^10^.

Nail involvement is likely to be encountered in the context of dactylitis, particularly in PsA, where it represents a very common feature. In this setting, the nail should not be regarded merely as a cutaneous appendage, but rather as a structure closely connected to the distal phalanx in the so-called nail–enthesis complex. Anatomically, the nail consists of a fibrous laminar structure, the nail plate, which overlies the nail bed and originates from the nail matrix. The nail matrix extends deeply to form the nail root, a highly vascularized region that is in continuity with both the distal interphalangeal joint capsule and the extensor tendon. Between the nail plate and the nail bed lies a rich neurovascular network embedded within connective tissue. This close anatomical relationship supports the link between nail and joint inflammation in PsA. Inflammation at the extensor tendon enthesis has also been demonstrated by MRI^21^. Nail changes may therefore reflect the involvement of adjacent structures—including the joint, enthesis, and tendons—which are also implicated in dactylitis. Accordingly, the coexistence of nail involvement and diffuse digit inflammation may help clinicians suspect PsA^22^.

The main nail changes in psoriasis may involve either the nail matrix or the nail bed. When the nail matrix is affected, the most common findings include pitting (small punctate depressions on the nail plate), leukonychia (white spots), and crumbling (nail plate fragility). When the nail bed is involved, subungual hyperkeratosis and detachment of the nail plate (onycholysis) can be observed^23^. These alterations not only reflect the involvement of specific components of the nail unit but may also help guide clinicians in the differential diagnosis with other nail manifestations seen in dactylitis of causes other than PsA (for example, the different pattern of nail involvement observed in sarcoid dactylitis)^24^.

### Etiopathogenetic considerations in dactylitis

Clinically, dactylitis presents as swelling of the entire digit. However, the underlying etiopathogenetic mechanisms may differ (inflammatory vs. non-inflammatory), as may the specific anatomical structures involved, which are not necessarily all affected in every case.

A well-established model of inflammatory dactylitis is that seen in PsA, where a multicompartmental process involves flexor tenosynovitis, soft tissue edema, synovitis, and enthesitis of pulleys and other structures in genetically predisposed individuals. An initial biomechanical insult may trigger an aberrant innate immune response with increased production of pro-inflammatory cytokines^25^.

In other forms, although a similar diffuse swelling of the digit is observed, there is no primary immune-mediated inflammation. In infectious forms, the primum movens is microbial invasion, while in sickle cell disease it is an ischemic/vaso-occlusive process; in both cases, the inflammatory response represents a secondary amplification^26,27^.

Similarly, in mechanically induced forms, such as pachydermodactyly, there are shared features with inflammatory dactylitis, including the role of mechanical stress as a trigger. However, while in inflammatory dactylitis mechanical stress promotes cytokine release, in pachydermodactyly it induces fibroblastic proliferation without significant immune involvement^28^.

#### Toward an etiopathogenetic classification

Using an etiopathogenetic approach, it is possible to identify three major categories of dactylitis^7^:

- non-inflammatory dactylitis (e.g., sickle cell dactylitis, pachydermodactyly, neoplastic dactylitis)
- inflammatory infectious dactylitis (e.g., tuberculous dactylitis, syphilitic dactylitis, blistering distal dactylitis)
- inflammatory non-infectious dactylitis (e.g., sarcoid dactylitis, SpA-related dactylitis, microcrystalline dactylitis)

#### Dactylitis associated with non-rheumatic disorders (Table 1): infectious etiologies

Tuberculous dactylitis is an extremely rare localization of Mycobacterium tuberculosis, accounting for only 1–3% of all tuberculosis cases. It occurs most frequently in children, typically affecting the phalanges of the hands, while multifocal involvement is exceptionally uncommon. Clinically, it presents with swelling and pain. Although rare, adult cases have also been reported, sometimes occurring in the absence of prior pulmonary symptoms. Notably, tuberculous dactylitis may also develop in patients receiving anti-TNF therapy despite negative baseline interferon-gamma release assays and normal chest radiographs. Radiographically, soft tissue swelling is observed, along with cystic cavities containing internal septations and cortical bone lytic lesions. Histological examination demonstrates caseous granulomas with epithelioid cells and Langhans-type giant cells. The characteristic “*spina ventosa”* appearance (resembling a “sail full of wind”) results from trabecular bone rarefaction and periosteal thickening. Management requires appropriate anti-tubercular pharmacotherapy^7,29^.

**Table 1:** Summary of dactylitis not related to rheumatologic disorders

Category	Condition	Incidence / typical age	Clinical features	Imaging findings
Non-inflammatory	Sickle Cell dactylitis (Hand–Foot Syndrome)	Children aged 6 months–2 years; more frequent in colder months	Painful swelling of hands/feet; vaso-occlusive crises	On X-ray: lytic lesions, bone necrosis, osteosclerosis
	Pachydermodactyly	Adolescents; males > females	Symmetrical, painless, elastic swelling of PIP joints; absence of synovitis	Soft tissue thickening only on US
	Celia disease	Rare	Painless finger swelling	Radiological bone features of osteomalacia
Neoplastic	Osteoid Osteoma	Benign bone tumor, common in children	Localized pain/swelling;	Radiolucent nidus on X-Ray
	Giant Cell Tumor of the Tendon Sheath	Most common benign tumor of the adult hand	Firm, elastic nodules adherent to tendons, Painless mass of soft tissue	Key findings on magnetic resonance, particularly on T2-weighted sequences, include ‘blooming’ on gradient-echo images due to hemosiderin deposition
	Paraneoplastic Dactylitis	Rare; associated with solid tumors (e.g., ovarian cancer)	Recurrent swelling as a paraneoplastic manifestation	
Infectious	Tuberculous Dactylitis (Spina ventosa)	1–3% of skeletal TB; children > adults	Gradual onset; Painful swelling of hands/feets; may occur without pulmonary symptoms	Soft tissue swelling (US); On X-ray: cystic cavities, lytic lesions, bone necrosis
	Non-tuberculous mycobacterial (M. marinum)	Associated with water exposure; incidence of 0.27 cases per 100,000 individuals	Cutaneous nodules/ulcers may progress to tenosynovitis/arthritis, often after trauma	Severe tenosynovitis, soft tissue edema, but findings are often nonspecific
	Congenital Syphilis	~2% of skeletal manifestations of syphilis	Bilateral, symmetrical involvement;	Periosteal reaction; minimal soft tissue swelling
	Yaws (Treponema pertenue)	Rare; endemic areas	Phalangeal lesions similar to syphilis	Findings similar to syphilis
	Blistering Distal Dactylitis (BDD)	Children	Volar (occasionally dorsal) vesicles; rapid onset; non-painful, non-pruritic	Fluid collection (anechoic on US) in the soft tissues without articular involvement
	Leprosy (Hansen’s Disease)	13% prevalence in large series (*n* ≈ 25,000)	Symmetrical joint involvement, Tenosynovitis, nodules, panniculitis, erythema nodosum leprosum	On US soft tissue edema, tenosynovitis (non-specific), On X-ray, lytic lesions, bone erosion
	Lyme Borreliosis	Rare	Chronic atrophic acrodermatitis; nodules mimicking dactylitis, swelling, and pain	On US soft tissue edema, tenosynovitis, and synovitis (non-specific)
Parasitic	Cutaneous Leishmaniasis	Extremely rare; 13 reported cases	Swelling, pain, granulomas with amastigotes; possible paronychia; exposure history	Soft tissue swelling on US radiography may show lytic lesions or periosteal reaction

Non-tuberculous mycobacterial dactylitis. Atypical mycobacteria linked to saltwater exposure (including domestic fish tanks), particularly Mycobacterium marinum, can lead to cellulitis that may progress to dactylitis if untreated. The soft tissue infection has an incidence of 0.27 cases per 100,000 individuals in the United States^30^. M. marinum penetrates the skin through direct injury; the incubation period ranges between 3 weeks and 9 months. Clinical presentations of Mycobacterium marinum infection are commonly observed in immunocompetent patients as single or limited cutaneous lesions, typically appearing as ulcerated nodules, and may also involve arthritis and tenosynovitis. In some cases, disseminated infections can occur, associated with pulmonary involvement and other systemic manifestations. Diagnosis is often delayed due to the long incubation period, making biopsy critical for confirmation. M. marinum is a naturally multidrug-resistant organism. Antibiotic therapy with clarithromycin, trimethoprim, and ciprofloxacin is considered effective, while combination therapy is required in cases of deeper infections^31,32^.

Syphilitic dactylitis is a rare manifestation of congenital syphilis. In this form, *Treponema pallidum* crosses the placenta, infiltrating sites of active endochondral ossification and impairing osteogenesis. It accounts for approximately 2% of bone manifestations of syphilis. The resulting osteochondritis may radiologically mimic tuberculous dactylitis; however, syphilitic lesions tend to be bilateral and symmetrical, with prominent periosteal involvement and minimal soft tissue swelling. Dactylitis may also occur in yaws disease, an uncommon tropical condition due to the *Treponema pertenue*, which results in periosteal changes indistinguishable from syphilitic dactylitis^7,33^.

**Fig. 2:**
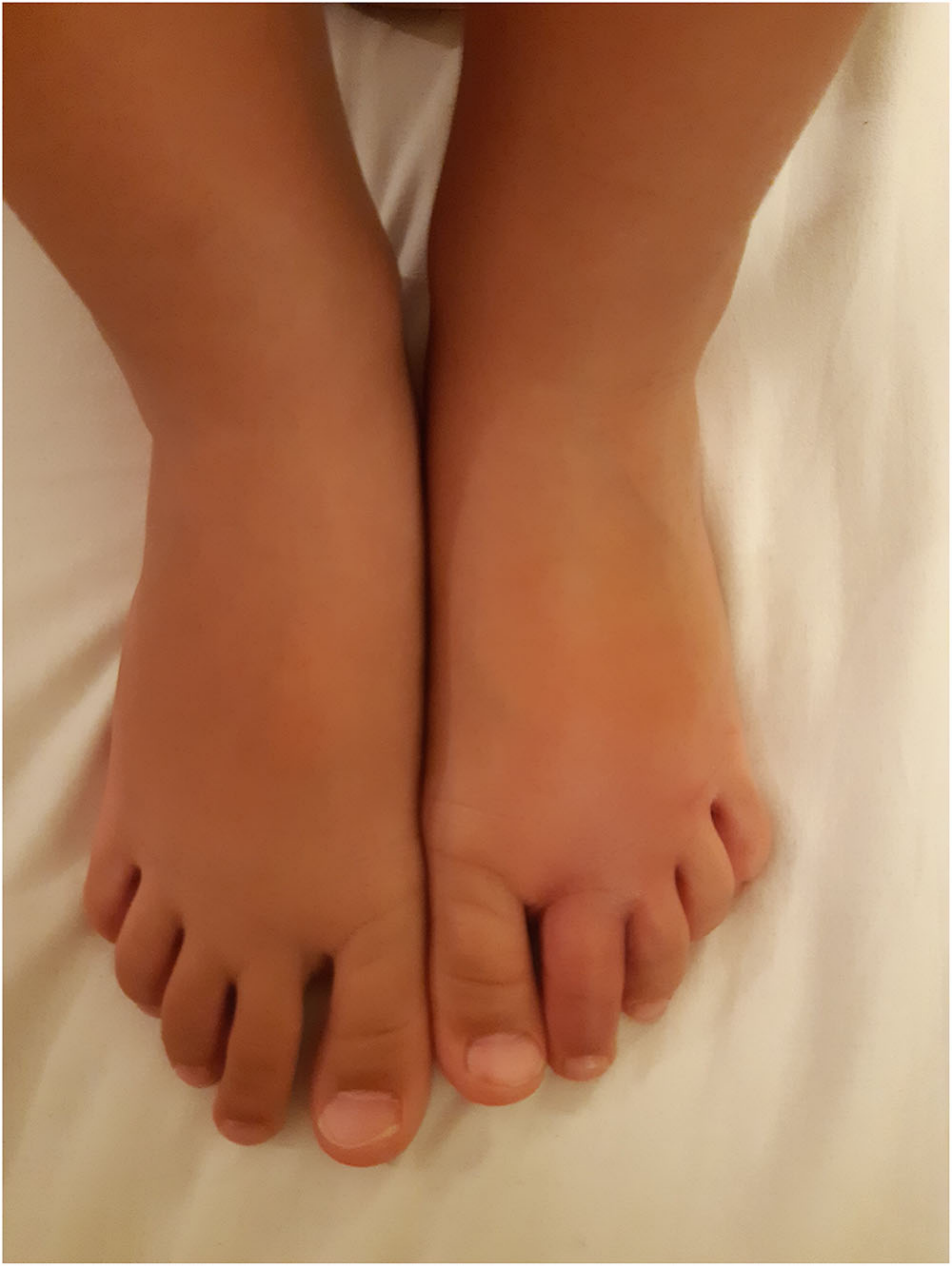
Blistering distal dactylitis. A 5-year-old girl presenting with acute swelling of the second toe of the left foot following an insect bite. Approximately 24 h after the insect bite, she developed fever and local erythema, consistent with an infectious dactylitis likely due to bacterial entry through a minor skin lesion. Complete resolution was achieved after antibiotic therapy.

Blistering distal dactylitis (BDD) is an infectious inflammatory condition, most frequently caused by *group A β-hemolytic Streptococcus* and typically observed in children. It involves the volar surface of the distal phalanges. Bacterial entry is often facilitated by small wounds or abrasions. Clinically, BDD presents with vesicles. Lesions usually develop within a few days, are neither painful nor pruritic, and recurrence is uncommon. Management consists of vesicle drainage combined with appropriate antibiotic therapy^7,34^ (Fig. 2).

Cutaneous Leishmaniasis can present with a broad spectrum of clinical features and, in rare cases, may manifest as inflammatory dactylitis. The epidemiology of this vector-borne disease needs to be considered. Eastern hemisphere leishmaniasis refers to cases occurring in the Old World, whereas Western hemisphere leishmaniasis is found in the Americas (New World). The key differences between Old World and New World leishmaniasis also relate to the causative species, clinical severity, and risk of mucosal involvement. New World cutaneous leishmaniasis shows greater variability in severity^35^. A detailed travel history to endemic areas is therefore essential. The onset of dactylitis is typically gradual and may be associated with marked skin scarring of the affected digit. Nail involvement is common, with the nail often appearing ischemic and, in some cases, shedding, particularly when nail bed involvement (paronychia) is present. The diagnostic work-up should consider patient occupational exposure to farming, along with specific polymerase chain reaction testing. Histopathology may reveal granulomatous histiocytic infiltrates containing Leishmania amastigotes. Standard treatment involves intramuscular administration of meglumine antimoniate (20 mg/kg/day) for at least one month. Miltefosine, a phospholipid analog, represents an alternative therapy^7,27^.

Leprosy (Hansen’s Disease), caused by *Mycobacterium leprae*, primarily affects the skin and peripheral nervous system but may also present with musculoskeletal manifestations in up to 70% of cases. The arthritis associated with leprosy typically involves the small joints of the hands and feet in an acute, symmetrical pattern. The swelling may result from inflammatory tenosynovitis and joint involvement, or (after treatment) from erythema nodosum leprosum—an acute inflammatory reaction to *M. leprae* antigens—characterized histologically by panniculitis with neutrophilic infiltrates and acid-fast bacilli. Diagnosis is based on dermatological and neurological examination, supported by skin smear and biopsy^36,37^.

Lyme Borreliosis (LB), a tick-borne infection caused by spirochetes of the *Borrelia burgdorferi* complex, may rarely present as inflammatory dactylitis. Weeks after infection, some patients develop chronic atrophic acrodermatitis (ACA), characterized by erythematous plaques and infiltrates over the extensor surfaces of the fingers, which may initially mimic dactylitis. In its chronic phase, ACA evolves into an atrophic, morphea-like skin appearance. Diagnosis of LB is based on the presence of ACA, skin biopsy that shows superficial and deep perivascular/interstitial lymphocytic infiltrates with plasma cells and eosinophils, serological detection of anti-*Borrelia* IgG, and clinical response to treatment. Management of dactylitis includes phenoxymethylpenicillin (1.5 million IU, three times daily for 10 days) or doxycycline (14–28 days) as first-line therapy^38^.

#### Dactylitis associated with non-rheumatic disorders (Table 1): non-infectious etiologies

Sickle cell anemia is a condition in which dactylitis can occur in children. The disease results from a mutation in the β-globin gene, leading to the production of hemoglobin S (HbS). Under hypoxic conditions, HbS polymerizes, causing erythrocytes to adopt a rigid, sickle shape. This morphological change promotes premature hemolysis and vaso-occlusion, which can affect joints and bones. Sickle cell dactylitis—also known as *hand-foot syndrome*—is a hallmark of homozygous sickle cell anemia. It results from localized infarction of the bone marrow of phalangeal bones. The transition from fetal hemoglobin (HbF) to HbS during the first year of life is a critical factor; lower HbF levels are associated with greater susceptibility to dactylitis. Environmental cold can precipitate painful crises. Dactylitis is considered a predictive factor for severe sickle cell complications. Radiographically, early findings include soft tissue swelling, radiolucent areas, lytic lesions, and focal osteosclerosis, producing a “worm-eaten” cortical appearance. Premature fusion of phalanges can result from necrosis of the epiphyseal center. The treatment is based on analgesia^7,26,39^.

**Fig. 3:**
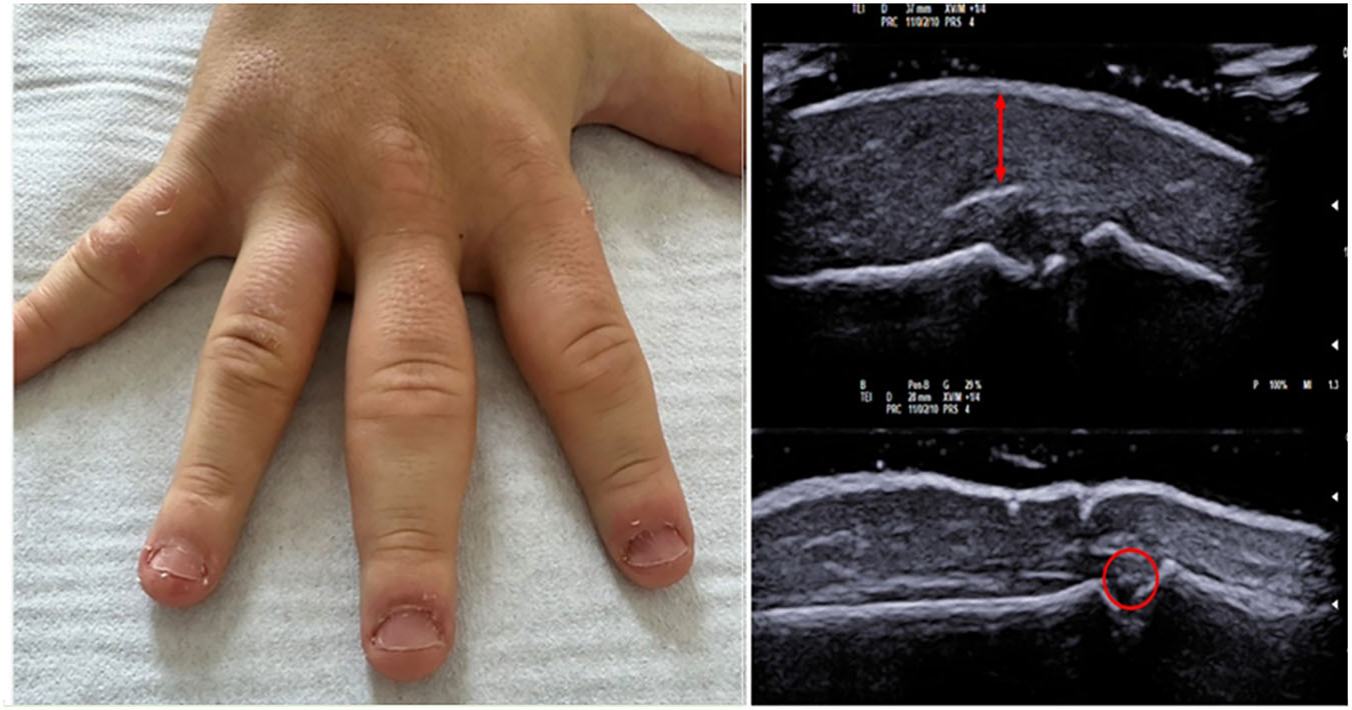
Dactylitis in pachydermodactyly. Left: Chronic, painless swelling of the third finger in an adolescent male. Right: Ultrasound images showing subcutaneous soft tissue thickening (arrow) and, on palmar longitudinal scan, absence of joint capsule distension or extensor tendon inflammation (circle).

Pachydermodactyly is a rare, benign form of digital fibromatosis characterized by progressive soft tissue thickening around the proximal interphalangeal joints. Swelling, usually symmetrical, most often affects adolescent males and is thought to be triggered by repeated mechanical trauma. The lesions are painless, elastic on palpation, and unaccompanied by joint synovitis. Imaging (US, magnetic resonance imaging-MRI, X-ray) reveals soft tissue thickening without involvement of deeper structures. Histology demonstrates marked hyperkeratosis, acanthosis, and dermal thickening. No effective medical therapy exists; surgical excision may be beneficial, and cessation of mechanical irritation can lead to spontaneous resolution^28^ (Fig. 3).

Osteoid osteoma is one of the most common benign bone tumors in children, typically affecting the long bones of the limbs but occasionally arising in unusual sites, such as the distal phalanges, where it may mimic dactylitis. Clinical features include local pain and swelling. Small lesions may be radiographically occult in early stages, making diagnosis challenging. When visible, the lesion appears as a radiolucent nidus with surrounding reactive osteosclerosis. Histologically, the nidus contains dilated vessels, osteoblasts, osteoid, and immature woven bone, encircled by sclerotic bone. Surgical removal is curative in most cases^40^.

Giant Cell Tumor of the Tendon Sheath (GCTTS) is one of the most common tumors of the hand in adults. It arises from the proliferation of synovial cells in tendon sheaths, producing elastic nodules intimately connected to the tendon. It typically occurs in middle-aged women and is generally slow-growing. This condition should be suspected when a painless mass appears in the volar region of the digit. In addition to physical examination, imaging techniques, such as plain radiographs or MRI, can aid in the differential diagnosis. Indeed, in the early stages, these nodules may displace adjacent structures and mimic the appearance of dactylitis^12,41^.

Paraneoplastic Dactylitis has been described in adults, sometimes as the first manifestation of an occult malignancy. Eviatar and Elkayam reported a patient with recurrent episodes of dactylitis in association with ovarian carcinoma^9^.

Dactylitis associated with celiac disease and vitamin D deficiency has also been reported in a 30-year-old Pakistani female patient with osteomalacia and celiac disease. The apparent dactylitis and the radiological bone changes resolved after treatment with a gluten-free diet and calciferol, as did the radiological bone features of osteomalacia^42^.

#### Dactylitis associated with rheumatic disorders (Table 2 and Fig. 4)

Psoriatic dactylitis is considered a hallmark of PsA. According to its clinical features, PsA dactylitis can be classified as active (hot) or chronic (cold). The former is characterized by swelling, pain, and, sometimes, redness; the latter by swelling without pain or other signs of inflammation. Recognition of dactylitis can facilitate early PsA diagnosis—within 12 months of symptom onset—and improved treatment^43,44^. It is a marker of disease severity associated with an increased risk of radiographic progression^45^. In addition to PsA, dactylitis can occur in other forms of SpA, particularly in reactive arthritis^45,46^. Peluso et al. demonstrated a prevalence of 15.38% in patients with enteropathic SpA, with higher frequency in those with Crohn’s disease and peripheral involvement, correlating with greater articular disease activity^47^. PsA-dactylitis is more comprehensively described in the following chapter.

**Table 2:** Summary of dactylitis related to rheumatologic disorders

Condition	Prevalence / Typical Age	Clinical Features	Imaging findings
Psoriatic dactylitis	Between 33 − 55%, but epidemiological data vary across studies	Painful or painless swelling; frequently asymmetric	Tenosynovitis, synovitis, and soft tissue edema; enthesitis with hypoechogenicity, thickening, and Power Doppler signal of the extensor tendon
Gout	About 5–9.6%	Polyarthritis; presence of tophi	severe soft tissue swelling, tenosynovitis, and tophi (hyperechoic masses on US)
Calcium hydroxyapatite deposition	Rare, rare case report	Acute pain due to calcific tendinitis; self-limiting	In the US, hyperechoic deposits in extra-articular structures (tendons) and periarticular calcifications
Sarcoid dactylitis	Rare epidemiological data vary across studies	May be asymptomatic; nail changes	On X-ray, lytic lesions; honeycomb pattern
Blau syndrome / EOS	Rare case reports	Camptodactyly; chronic, fixed, painless	Synovial hypertrophy
Calcium pyrophosphate deposition disease dactylitis	Rare case reports	acute, diffuse finger swelling and functional impairment; achieved rapid remission following a short course of colchicine;	In the US, synovial hypertrophy, severe soft tissue swelling, tenosynovitis

Microcrystalline dactylitis has been described in microcrystalline arthropaties.

**Fig. 4:**
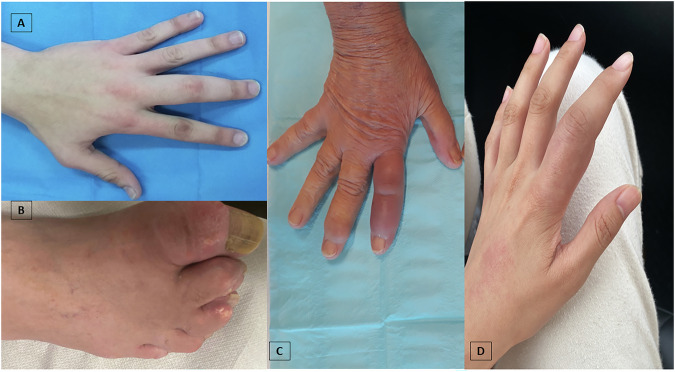
Dactylitis related to rheumatologic disorders. **A** Inflammatory dactylitis in a patient with psoriasis involving the elbows and knees, presenting with pain and functional limitation of the third finger of the left hand. The dactylitis showed a rapid and marked response to ultrasound-guided corticosteroid injection into the flexor tendon sheath. **B** Chronic dactylitis in a 70-year-old patient with known gouty arthritis, presenting with multiple episodes of diffuse swelling of the first toe of the right foot. The patient was not receiving therapy due to poor compliance. The clinical picture was consistent with tophaceous deposition, also involving other digits. **C** Acute dactylitis in an 82-year-old patient with known calcium pyrophosphate deposition (CPPD) disease arthritis, presenting with first episode of diffuse swelling of the second finger of the right hand. Other causes were excluded. The clinical picture was consistent with crystal-induced inflammatory dactylitis and showed a good response to a course of colchicine. **D** Dactylitis in a middle-aged woman with sarcoidosis, presenting with diffuse swelling and pain of a finger, associated with respiratory symptoms (dry cough and dyspnea) and peripheral arthritis, with excellent response to anti–TNF-α therapy.

Gout is a form of inflammatory arthritis characterized by precipitation of sodium monourate (MSU) crystals in joints and soft tissues following chronic hyperuricemia. It has been reported in 5–9.6% of patients with gout^48^. Dactylitis was observed in the polyarticular form and appeared to be a predictor of disease severity. In fact, patients with dactylitis exhibited a longer disease duration, greater joint involvement, higher serum uric acid levels, more tophi, higher ESR values, and fulfilled more of the American College of Rheumatology/European League Against Rheumatism (ACR/EULAR) gout classification criteria beyond crystal identification^49,50^. Gout was confirmed at the time of presentation by the identification of MSU crystals, either aspirated from soft-tissue tophi of the affected digits or from the adjoining joints.

Calcium hydroxyapatite deposition disease may lead to crystal deposition in tendons (referred to as calcific tendinitis), a common condition that may be asymptomatic or cause acute or chronic symptoms. Calcific tendinitis has been documented in nearly every tendon, with the shoulder being the most frequently affected site (approximately 60%), followed by the tendons of the hip, fingers, toes, and wrists^51–54^. Acute calcific tendinitis affecting the fingers has been reported and should be considered a potential cause of hand dactylitis. Acute, self-limiting resorptive episodes typically present with sudden, intense pain and localized inflammation, with symptoms generally resolving within 4 to 7 days^55^.

Calcium pyrophosphate deposition disease (CPPD). Dactylitis is not a recognized clinical feature of calcium pyrophosphate deposition (CPPD) disease. However, Girolimetto et al. described two elderly women who developed acute dactylitis attributed to CPPD following a prolonged period of disease quiescence. The diagnosis of CPPD had previously been established by radiographic evidence of chondrocalcinosis and identification of crystals in synovial fluid. Both patients subsequently presented with acute, diffuse finger swelling and functional impairment consistent with dactylitis. Alternative diagnoses were excluded, and one patient achieved rapid remission following a short course of colchicine^56^.

Sarcoid dactylitis is a rare manifestation of chronic sarcoidosis, occurring in fewer than 1% of patients. It is characterized by granulomatous inflammation and lytic bone lesions with a honeycomb aspect of trabecular bone in the radiographic appearance. Non-caseating granulomas are found within the phalanges and periarticular soft tissues. Sarcoid dactylitis may present as the only musculoskeletal manifestation. Skin lesions, particularly lupus pernio, frequently coexist, and dactylitis in this context is associated with a worse prognosis in multisystemic disease^57–59^.

Sarcoid dactylitis is often associated with dystrophic nail changes. The nail in the affected finger appears brittle, thin, clubbed with subungual keratosis, longitudinal ridging, paronychia, and brown discolouration of the nail bed. The dactylitis in sarcoid can be restricted to part of the finger overlying the affected phalanx or phalanges. A plain X-ray is needed for the affected digit and the whole hand to look for the diagnostic bone changes of sarcoidosis in the phalanges^24^.

Dactylitic involvement in pediatric forms of sarcoidosis is described in Blau syndrome (BS) and early-onset sarcoidosis (EOS)^60^. In these disorders, caused by NOD2 mutations, the same granulomatous process that in adults produces dactylitis instead leads over time to camptodactyly, a fixed and fibrotic contracture of the digits^61^. Dactylitis reflects acute, reversible inflammatory activity, whereas camptodactyly testifies to chronic, structural remodeling and damage^60,61^.

BS was first described in 1985, and together with EOS forms the inherited and sporadic manifestations of the same disorder. The classic triad is granulomatous arthritis, dermatitis, and uveitis, typically arising in the first years of life. Over time, the persistence of granulomatous inflammation remodels periarticular tissues, inducing capsular fibrosis and tendon shortening that culminate in camptodactyly. Unlike sarcoid dactylitis, which is painful, swollen, and reversible, Blau/EOS camptodactyly is painless, fixed, and permanent^62–64^. Histology reveals non-caseating granulomas with epithelioid histiocytes, multinucleated giant cells, and dense lymphocytic coronas. Immunohistochemistry demonstrates prominent IFN-γ, IL-6, and IL-17 expression, consistent with pathogenic activation of both Th1 and Th17 pathways^62,64^. Clinically, BS /EOS camptodactyly is bilateral, symmetric, and predominantly affects the proximal interphalangeal joints. Unlike erosive arthritides, joint surfaces remain preserved, and destructive changes are rarely observed on radiographs. In BS/EOS, US and MRI show synovial hypertrophy, Doppler-positive tenosynovitis, and fibrotic remodeling of periarticular tissues. The therapeutic approach to camptodactyly in BS/EOS is early and sustained suppression of granulomatous inflammation by corticosteroids and DMARDs to prevent irreversible contractures and other manifestations. Once established, camptodactyly is essentially refractory to systemic therapy, and orthopedic correction provides at best partial functional benefit^62^.

### Psoriatic dactylitis

The estimated prevalence of dactylitis in PsA is between 33 an 55%, with approximately 70% of cases occurring at presentation^25^. It is a marker of disease severity associated with an increased risk of radiographic progression^45^. Data from the Toronto PsA cohort indicate that radiographic progression in hot dactylitis is common after an average disease duration of eight years^65^. Therefore, early recognition of dactylitis is crucial for initiating targeted therapy and preventing long-term joint damage^66^. It affects feet more often than hands, in an asymmetrical distribution, with the right side more frequently involved, supporting the hypothesis that trauma may act as a trigger for PsA^65^. The fourth toe of the right foot is the digit most frequently involved.

Its etiopathogenesis remains to be established, but it is likely that, in individuals with a genetic predisposition, an initial biomechanical insult associated with other factors may lead to an aberrant innate immune response with overproduction of pro-inflammatory cytokines. A study by McGonagle et al. proposed a pathogenetic model of dactylitis during PsA. This was based on human and experimental models. Cells of innate immunity (neutrophils, macrophages, and γδ T-cells), activated by micro-trauma and other factors, release pro-inflammatory cytokines, triggering local inflammation and inducing an over-activation of adaptive immunity with infiltration of T cells into soft tissue. Animal pathogenetic models have confirmed a central role of TNF and the IL23/IL-17- cytokine axis in the pathogenesis of dactylitis^25^.

#### Clinical assessment of psoriatic dactylitis

The development of new treatments for PsA has required the introduction of measurement tools to quantify dactylitis. The Leeds Dactylitis Index (LDI), proposed by Helliwell et al., is based on digital circumference and tenderness, comparing the affected digit with its contralateral counterpart (if unaffected). A ≥ 10% difference in circumference defines dactylitis. Reference tables are provided for cases of symmetric involvement of two digits. This method has shown good inter- and intra-observer reliability but may be limited in obese patients, where excess fatty tissue hampers measurement^25,67^.

### Role of imaging in dactylitis

#### Ultrasound

The advent of high-frequency probes in modern US systems has enabled highly detailed imaging of small and superficial anatomical structures, including the digits. For the accurate assessment of dactylitis, the use of 7.5–13.5 MHz probes is recommended; although higher frequencies (up to 22 MHz) can further improve resolution, particularly for visualizing the pulleys^1,66,68,69^.

**Fig. 5:**
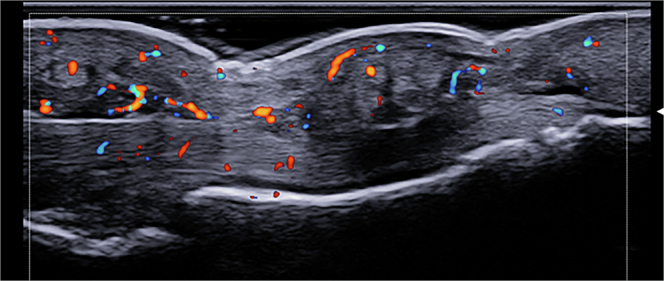
Ultrasound features of active dactylitis. Gray-scale longitudinal reconstruction of the volar aspect of a finger affected by active dactylitis, obtained through multiple scans, showing thickened, iso-hypoechoic subcutaneous edema and distension of the flexor tendon sheath surrounding the tendon. Power Doppler imaging demonstrates a scalloped appearance of the flexor tendon in regions lacking pulley support, with marked intra-tendinous hypervascularisation. (PD power Doppler).

In the US, dactylitis typically presents as a combination of synovial and extra-synovial inflammatory changes, including joint synovitis, enthesitis, flexor tendon tenosynovitis, and subcutaneous tissue edema (STO). All ultrasonographic abnormalities observed in dactylitis are defined according to OMERACT definitions^46,70,71^ (Table 3 and Fig. 5).

**Table 3:** Ultrasound Assessment of Dactylitis

Anatomical Structure	Normal Ultrasound Appearance	Pathological Findings in Dactylitis
Synovium	Hypoechoic thin layer within capsule; no PD signal	Thickened hypoechoic tissue, non-displaceable, poorly compressible, with or without PD activity (synovitis)
Flexor Tendon Sheath	Thin, hyperechoic line surrounding the tendon	Hypoechoic/anechoic halo, sheath distension (‘festooned appearance’), ± PD signal (tenosynovitis)
Extensor Tendon	Fibrillar echotexture, centrally located under the extensor hood	Altered echogenicity, displacement outside the hood (sagittal band rupture), increased PD signal
Extensor Hood & Sagittal Bands	Thin echogenic band; transverse ‘seagull’ sign	Thickening, discontinuity, PD signal; subluxation of tendon
Central Slip & Enthesis	Continuous fibrillar structure inserting on middle phalanx	Enthesitis with hypoechogenicity, thickening, PD signal; cortical irregularity or avulsion
Peritendinous Soft Tissues (Extensor)	Uniform echotexture, no PD activity	Hypoechoic swelling surrounding the tendon (PTI), ± PD signal
Subcutaneous Tissues	Normal echogenic subcutaneous fat	Diffuse swelling (STO), increased PD signal from finger pad to MCP joint
Pulleys (A1–A5, C1–C3)	Linear anisotropic band ≤0.60 mm; hyperechoic volar portion	Increased thickness, hypoechogenicity, partial or complete rupture; bowstring deformity (A2/A4), trigger finger (A1)
Collateral Ligaments	Hyperechoic fibrillar bands at lateral joint margins	Thickening, partial rupture, altered echotexture, instability on dynamic scan

Extracapsular structures, including the extensor hood, are key contributors to the pathogenesis of psoriatic arthritis. The extensor hood can be visualized as a thin echogenic band, with the characteristic “seagull sign” in transverse view. These structures, together with the central slip enthesis and adjacent synovium, form the synovio-entheseal complex (SEC)^12,46,72^. Further studies showed that extracapsular involvement correlated more strongly with symptomatic (“hot”) dactylitis than with asymptomatic (“cold”) dactylitis, the latter more frequently presenting with synovitis^73^. In early, untreated PsA, it has been observed that hot dactylitis was most frequent in toes (particularly the second and fourth)^66^.

#### Ultrasonographic assessment in PsA

Imaging-based tools have been developed to provide a more comprehensive evaluation of dactylitis. DACTOS score is currently the most widely adopted tool for evaluating involvement of the anatomical components in ultrasound dactylitis. It is sensitive to change and reliable in daily practice^74^. The DACTylitis glObal Sonographic (DACTOS) score integrates ultrasonographic findings into a global score from 0 to 25. It has demonstrated high reliability among expert ultrasonographers, although subcutaneous edema and peritendinitis showed slightly lower concordance^75^. The OMERACT Global Ultrasound Score for Dactylitis (GLOUDAS), developed by Naredo et al., assesses the main anatomical structures involved in psoriatic dactylitis. Through Delphi consensus, 12 digital entheses were selected for the final version of the score. Intra-observer reliability was excellent^76^. Zhao et al. used the DACTOS score to identify subclinical dactylitis on US, finding an association with a more severe PsA phenotype.

#### Magnetic resonance imaging

MRI offers unparalleled visualization of soft tissues, joints, and entheses, providing diagnostic insights not achievable with other imaging modalities. Gadolinium-based intravenous contrast is routinely used to enhance the detection of abnormalities and to distinguish effusion from synovitis.

A recommended MRI approach for dactylitis includes T1-weighted, T2-weighted, and short tau inversion recovery (STIR) sequences^77^. Bone marrow edema appears as poorly defined subcortical hyperintensity on fluid-sensitive sequences and is often accompanied by extensive soft tissue edema with a “honeycomb” pattern on T2-weighted images^19,78,79^.

Previous MRI studies in PsA have shown that dactylitis is primarily associated with flexor tendon tenosynovitis, while joint effusion is typically less prominent^80^. In 2015, Tan et al. described inflammation of small entheses associated with tendon pulleys—termed functional entheses—which are subject to repetitive mechanical stress. This led to the concept of “digital polyenthesitis” as a mechanism underlying dactylitis in PsA^81^. However, the digital polyenthesitis model does not fully explain all forms of dactylitis; current evidence suggests that dactylitis pathophysiology is heterogeneous, involving both entheseal and non-entheseal structures, with varying contributions depending on the stage of the disease^81^.

MRI can also demonstrate similar patterns of tenosynovitis in traumatic conditions, interpreted as a Koebner-like phenomenon^82^.

MRI can also contribute to the assessment. The Psoriatic Arthritis Magnetic Resonance Imaging Hand Scoring System (PsAMRIS-H), developed by OMERACT, can identify features of dactylitis and evaluate treatment response with high intra- and inter-reader reliability^45,83^. Although MRI is highly accurate in detecting tenosynovitis, US is useful for identifying insertional enthesitis and has been used to visualize enthesitis at the deep flexor tendon insertions in PsA^84,85^.

Although most imaging data derive from PsA, imaging findings (US, X-ray, and MRI) may also be relevant in dactylitis of other etiologies, including microcrystalline diseases, where they can contribute to the differential diagnosis.

MRI findings showed that digit involvement in gouty dactylitis can result from different pathological mechanisms: a true inflammatory condition (characterized by arthritis, tenosynovitis, and soft tissue edema) as well as a non-inflammatory process involving accumulation of hyperintense material circumferential (possibly MSU) in the subcutaneous tissue of the affected digit, known as dactylopathy^48,86^.

US and MRI may show acute synovitis of the flexor sheath in microcrystalline diseases^56^. On MRI, periarticular calcifications appear as low-signal-intensity deposits on both T1- and T2-weighted images and may be overlooked. MRI findings of surrounding soft-tissue and bone edema can lead to misdiagnosis as a more aggressive pathology, especially if MRI is the initial imaging modality used^52^.

### Treatment of PsA dactylitis

The evidence for the treatment of dactylitis in PsA remains limited, as it is most often considered a secondary outcome in clinical trials. Only a few studies have evaluated dactylitis as a primary endpoint. The GO-DACT trial, which compared methotrexate (MTX) monotherapy with a combination of MTX and golimumab in patients with dactylitis, demonstrated that the combination of a conventional disease-modifying antirheumatic drug (cDMARD) and golimumab was more effective than MTX alone^87^. An Italian multicentre real-life study showed that apremilast is efficacious in PsA dactylitis^88^. Finally, small-molecule therapies, such as Janus kinase (JAK) inhibitors, have also shown efficacy in dactylitis. However, outcome assessment methods for dactylitis in RCTs remain highly heterogeneous^89^.

NSAIDs are the first-line treatment for isolated dactylitis. In the EULAR recommendations, there is no specific indication for dactylitis. Their suggestion is to treat this manifestation according to its association with other manifestations (polyarthritis or others) and, in case of isolated dactylitis, to use the same approach of oligoarthrits^90^. In the GRAPPA recommendations, after NSAIDs failure, DMARDs, including MTX, are considered indicated^91^.

Glucocorticoid injections represent an efficacious therapeutic option. Common preparations include triamcinolone acetonide, triamcinolone hexacetonide, methylprednisolone acetate, dexamethasone sodium phosphate, and betamethasone sodium phosphate. Injections should be performed at a 45° angle to the skin—preferably under US guidance—to avoid tendon injury. The out-of-plane US-guided technique is often preferred in clinical practice, as it reduces procedural risks; combining this with a local anesthetic to “clean” the needle track can further reduce the risk of subcutaneous atrophy^92^. Girolimetto et al. reported that corticosteroid injection was effective in 90% of patients with dactylitis at three months post-procedure, with a significant reduction in extracapsular inflammation^93^.

In clinical practice, patients unresponsive to NSAIDs or corticosteroid injections may be treated with csDMARDs, especially MTX. In refractory cases, with relevant clinical impact, TNFi, or IL- inhibitors (IL-12/23i or IL-17i or IL-23i) or phosphodiesterase-4 inhibitor should be considered^25,92,94^.

## Conclusion

Dactylitis represents a clinical entity of considerable interest for the rheumatologist. In addition to being a marker of common diseases, such as PsA and other SpAs, it may also represent a non-specific sign of a wide range of disorders, as outlined in this review.

A thorough understanding of the anatomical structures composing the digit, and therefore involved in the development of dactylitis, is essential. In this context, advances in imaging, particularly MSK-US, have a pivotal role in improving diagnostic accuracy. The integration of clinical assessment with the imaging modalities currently available to clinicians allows correct diagnoses in most cases and the implementation of more appropriate therapeutic strategies.

In conclusion, dactylitis should be regarded as a multifaceted clinical sign that extends beyond PsA and other forms of PsA and that requires clinicians to consider a number of alternative etiologies. Ongoing advances in imaging are expected to further enhance our understanding of its pathogenesis and facilitate earlier and more targeted therapeutic interventions

## Supplementary information

Transparent Peer Review file
