# Supplementary material for: The wider implications of dactylitis beyond psoriatic arthritis
Source: Commun Med (Lond). 2026 Jul 24;6:412. doi: 10.1038/s43856-026-01730-3 (PMC13400733; doi:10.1038/s43856-026-01730-3)
Supplement: Supplementary file 1 — Transparent Peer Review file [file 43856_2026_1730_MOESM1_ESM.pdf]

nature portfolio

Peer Review File

The wider implications of dactylitis beyond psoriatic arthritis

Corresponding Author: Dr Philip Helliwell

This file contains all reviewer reports in order by version, followed by all author rebuttals in order by version.

Version 0:

Reviewer comments:

Reviewer #1

(Remarks to the Author)
The authors present an extensive narrative review of dactylitis covering the anatomy, etiopathogenesis, differential diagnoses, imaging features, prognostic implications, and treatment, with a focus on PsA. It's overall well-written. Nonetheless, the extensive nature of this review itself can be a deterrent for a comprehensive read. Though the title is 'Beyond PsA', the authors have focused primarily on spondyloarthritis, including its treatment. There is a fair bit of redundancy in this review that needs to be addressed, especially integration across the sections. I would invite the authors to revise the review, focusing primarily on the structure, redundancy, and proportionality of the content.

Here are some major comments for the authors to consider:
1. Kindly consider reducing the length of the review, focusing on the practical issues. It's very loaded in the current form and can easily distract the reader.
2. The historical background is good, but it may not be relevant to the targeted readers and can be abridged.
3. The anatomical considerations section is well written, but again, too extensive, with some redundancy. The line drawings are excellent and self-explanatory. The detailed description of pulleys in the text may not be required, especially for this targeted readership. It would be better if the authors streamlined the descriptive anatomy and focused on structures relevant to inflammation in PsA.
4. How much does the 'digital polyenthesitis' concept play a role in defining inflammatory and mechanical dactylitis? I would suggest that the authors focus on the mechanistic core of dactylitis and polyenthesitis.
5. What differentiates inflammatory and non-inflammatory dactylitis mechanistically? A bit of detail on the shared and divergent mechanistic theories across these etiologies will be helpful.
6. Flexor tenosynovitis is described both in the etiopathogenesis and imaging sections.
7. The table on 'summary of dactylitis not related to rheumatologic disorders' is excellent and gives ample room to concise the text around that. It would be great if the authors could manage some clinical images of non-inflammatory dactylitis.
8. Dactylitis in the pediatric age group can be concise. It is information-overloaded in its current form.
9. Imaging details can be grouped together. For instance, in the section on calcium hydroxyapatite deposition disease, the authors have mentioned imaging. This could be moved to the imaging section to maintain uniformity.
10. Dactylitis in spondyloarthritis is generally mentioned as active and chronic, and not acute and chronic.
11. I would describe clinical and imaging scoring systems separately. The authors have clubbed LDI and DACTOS together as measurement tools, which could confuse the reader.
12. Could the authors include a reference for the statement on using 7.5-13.5 MHz probes for dactylitis assessment in the ultrasound section?
13. Again, the ultrasound section is too long and includes a table.
14. The authors claim that MRI assessment can be challenging due to the complex anatomy of the structures involved. The concept of polyenthesitis was based on a high-resolution MRI study. So, this sentence doesn't make sense.
15. In the MRI section, what do the authors mean by this sentence? "The examination typically begins with whole-hand imaging to detect all abnormalities at the highest available spatial resolution, followed by targeted imaging of the affected digit with a reduced field of view." This can only confuse the reader.
16. The authors have recommended an MRI protocol for dactylitis. What's the reference for this? Also, what's the reference to mention that the ultrasound is more sensitive than MRI for dactylitis imaging?

Minor comments:
1. There are some minor grammatical errors in the sentences that need to be addressed.

Reviewer #2

(Remarks to the Author)
The manuscript reviews many aspects of dactylitis as a symptom of diverse medical conditions. It is a well-written and interesting paper. I have several minor suggestions for the authors:
- line 76. Please, re-check the grammar
- line 242. The phrase 'bone expansion due to destruction' is unclear. Please, explain
- line 247. Dactylitis due to non-tuberculous mycobacteria, particularly M. marinum, is not uncommon at all and should be discussed in more depth, better in a separate sub-chapter, including epidemiology and tips for diagnosis
- line 333. Please, add when to suspect and how to diagnose GCTTS
- line 347. Non-tuberculous mycobacterial dactylitis should be added to the table
- lines 115, 120, 354, 423 refer to the prevalence of PsA-dactylitis. Why is it different every time?
- line 367. Should 'higher' be used in place of 'elevated'?
- line 367. ACR criteria for what?
- line 369. Please change to 'aspirated from soft-tissue tophi.'
- lines 120 and 391 - again, the mentioned prevalence of sarcoid dactylitis is different
- lines 424-426. Please, re-check the grammar
- lines 428. Please, discuss the point of view that dactylitis is a subtype of enthesitis or ligamentitis.
- lines 437-440 probably should appear at line 351, after the citation 33.

Reviewer #3

(Remarks to the Author)
Comments
1. A main component of every digit is the nail. It has been completely overlooked in the manuscript, both in the anatomy and various causes of dactylitis.
2. Another cause of dactylitis is coeliac disease combined with vit D deficiency. A 30-year-old Pakistani female patient with osteomalacia and coeliac disease presenting with dactylitis (Jawad et al. Osteomalacia and coeliac disease presenting as isolated dactylitis Annals of the Rheumatic Diseases, 1988; 47, 692-694). The apparent dactylitis and the radiological bone changes resolved after treatment with a gluten free diet and calciferol, as did the radiological bone features of osteomalacia.
3. Sarcoid dactylitis is often associated with dystrophic nail changes. The nail in the affected finger appears brittle, thin, clubbed with subungual keratosis, longitudinal ridging, paronychia and brown discolouration of the nail bed. (Jawad AS. Sarcoid dactylitis with nail involvement. JRCPE 2022). https://doi.org/10.1177/1478271522111305. The nail changes need to be compared to the nail changes in psoriasis. The dactylitis in sarcoid can be restricted to part of the finger overlying the affected phalanx or phalanges. A plain X-ray is needed for the affected digit and the whole hand to look for the diagnostic bone changes of sarcoidosis in the phalanges.
4. TB dactylitis. The onset is often gradual in comparison to BDD. We have seen 2 patients presenting with TB dactylitis while on anti TNF therapy for Crohn's disease in spite of negative gamma interferon test and a normal chest X-ray prior to starting therapy. The reader needs to be alerted to this observation.
5. If there are features specific to the type of dactylitis such as the nail changes in sarcoid and psoriatic dactylitis, they need to be described.
6. A table comparing the clinical and imaging findings of dactylits in common condition would be useful to the reader.
7. Leishmaniasis: The epidemiology of this vector-born disease need to be included. Eastern hemisphere leishmaniasis refers to Leishmaniasis found in Africa, Europe, Asia, and Oceania (previously referred to Old World leishmaniasis). Western hemisphere leishmaniasis refers to the disease found in the Americas (New World leishmaniasis). Travel history to endemic areas needs to be emphasized. The onset of dactylitis in leishmaniasis is gradual and is characterised by excessive scarring of the skin of the digit. Nail changes are common and often the nail looks ischemic and it may fall.
8. More clinical photos are needed especially of psoriatic and sarcoid dactylitis.
9. The abstract needs to be improved stating its importance in commonly seen conditions such as psoriasis, SpA and sarcoidosis.
10. Figure 1. There are numbers of the diagram but not explained in the legend.
11. Figure 2. The central slip looks brown rather than red as mentioned in the legend.
12. There is no proper Discussion and the concluding statement can be improved and be more informative.
13. The numbering the various sections of the manuscript does not look logical.

Version 1:

Reviewer comments:

Reviewer #1

(Remarks to the Author)
I would like to thank the authors for carefully reviewing the comments and addressing them. The review has improved considerably. I don't have any further comments.

Reviewer #2

(Remarks to the Author)
The manuscript was properly revised.

Reviewer #3

(Remarks to the Author)
I thank the authors for revising the manuscript. It is very informative and clearly written. I have no negative comments.

Open Access This Peer Review File is licensed under a Creative Commons Attribution 4.0 International License, which permits use, sharing, adaptation, distribution and reproduction in any medium or format, as long as you give appropriate credit to the original author(s) and the source, provide a link to the Creative Commons license, and indicate if changes were made.
In cases where reviewers are anonymous, credit should be given to 'Anonymous Referee' and the source.
The images or other third party material in this Peer Review File are included in the article’s Creative Commons license, unless indicated otherwise in a credit line to the material. If material is not included in the article’s Creative Commons license and your intended use is not permitted by statutory regulation or exceeds the permitted use, you will need to obtain permission directly from the copyright holder.
To view a copy of this license, visit https://creativecommons.org/licenses/by/4.0/

Reviewers' comments

Reviewer #1 (Remarks to the Author): *"The authors present an extensive narrative review of dactylitis covering the anatomy, etiopathogenesis, differential diagnoses, imaging features, prognostic implications, and treatment, with a focus on PsA. It's overall well-written. Nonetheless, the extensive nature of this review itself can be a deterrent for a comprehensive read. Though the title is 'Beyond PsA', the authors have focused primarily on spondyloarthritis, including its treatment. There is a fair bit of redundancy in this review that needs to be addressed, especially integration across the sections. I would invite the authors to revise the review, focusing primarily on the structure, redundancy, and proportionality of the content".*

Response: We thank the reviewer for the insightful comments and for appreciating the comprehensiveness of our work. In response to these suggestions, we have revised the structure of the manuscript to make the text more concise and reducing redundancy across sections. In particular, we have streamlined the anatomical section, which we agree could appear overly detailed and potentially dispersive. Regarding the integration between sections, we have carefully revised the manuscript to enhance coherence throughout. From a rheumatological perspective, dactylitis is most commonly recognized as a hallmark feature of psoriatic arthritis. For this reason, we have provided a more in-depth discussion of the diagnostic and therapeutic aspects specifically related to this condition. We hope that these revisions have improved the clarity, balance, and overall structure of the manuscript

Here are some major comments for the authors to consider:

Reviewer #1, Comment 1: *Kindly consider reducing the length of the review, focusing on the practical issues. It's very loaded in the current form and can easily distract the reader.*

Response: Thank you for this suggestion. We have revised the manuscript. Several sections have been shortened, and redundant content has been removed to avoid overloading the reader. We hope these changes have made the review more focused and accessible. For clarity, we have also provided a marked-up version of the manuscript using track changes.

Reviewer #1, Comment 2: **"***The historical background is good, but it may not be relevant to the targeted readers and can be abridged"*

Response: Thank you for this valuable comment. We have revised the manuscript by substantially shortening the historical background, retaining only key elements necessary to contextualize the definition and clinical relevance of dactylitis.

Revised Text:

The earliest description of dactylitis is attributed to Avicenna in the 11th century, who reported finger swelling in the context of infectious diseases. The classical definition of dactylitis, provided in 1998 by Rothschild BM et al., describes it as a uniform swelling of the digit.[1] Earlier clinical observations also highlighted the contribution of joint and tendon sheath inflammation to the characteristic "sausage-like" appearance.[2] Initially, dactylitis was not considered a clinically distinct or relevant feature of PsA[3]. Its relevance was clearly established with the CASPAR criteria in 2006, where dactylitis (current or past) emerged as a highly specific feature of PsA, observed in 54% of cases.[4] Dactylitis, however, may be due not only to PsA or other inflammatory conditions, but also to a variety of other disorders including trauma or congenital deformities[5].

Epidemiological data vary across studies and are influenced by differences in patient populations, with reported prevalence rates of 39% in PsA, 17% in sarcoidosis, 9.5–12% in other SpAs, and 5–9.6% in gout.[6] *Furthermore, dactylitis may represent the manifestation of a neoplastic process (particularly osteoid osteoma) or a paraneoplastic syndrome.*[7,8]

Reviewer #1, Comment 3: **"***The anatomical considerations section is well written, but again, too extensive, with some redundancy. The line drawings are excellent and self-explanatory. The detailed description of pulleys in the text may not be required, especially for this targeted readership. It would be better if the authors streamlined the descriptive anatomy and focused on structures relevant to inflammation in PsA"*

Response: Thank you for this insightful comment. We have revised the anatomical considerations section by reducing redundancy. In particular, we have shortened the detailed description of pulley anatomy. In addition, we have simplified the figures to further enhance clarity and avoid unnecessary complexity.

Revised Text:

In addition to bones and synovial joints, in each digit can be identified three main regions: the dorsal region, which houses the extensor apparatus; the volar region, which contains two flexor tendons anchored to cortical bone through a pulley system; and the lateral region, consisting of collateral ligaments that contribute to joint stability.[10] *Classical anatomical classifications, such as those proposed by Verdan, Kleinert and Quílez Caballero, allow precise localization of tendon injuries and extensor structures.*[11,12]

A thorough knowledge of digital anatomy is essential, as specific structures represent key targets of inflammation in diseases such as PsA. (Figure 1)

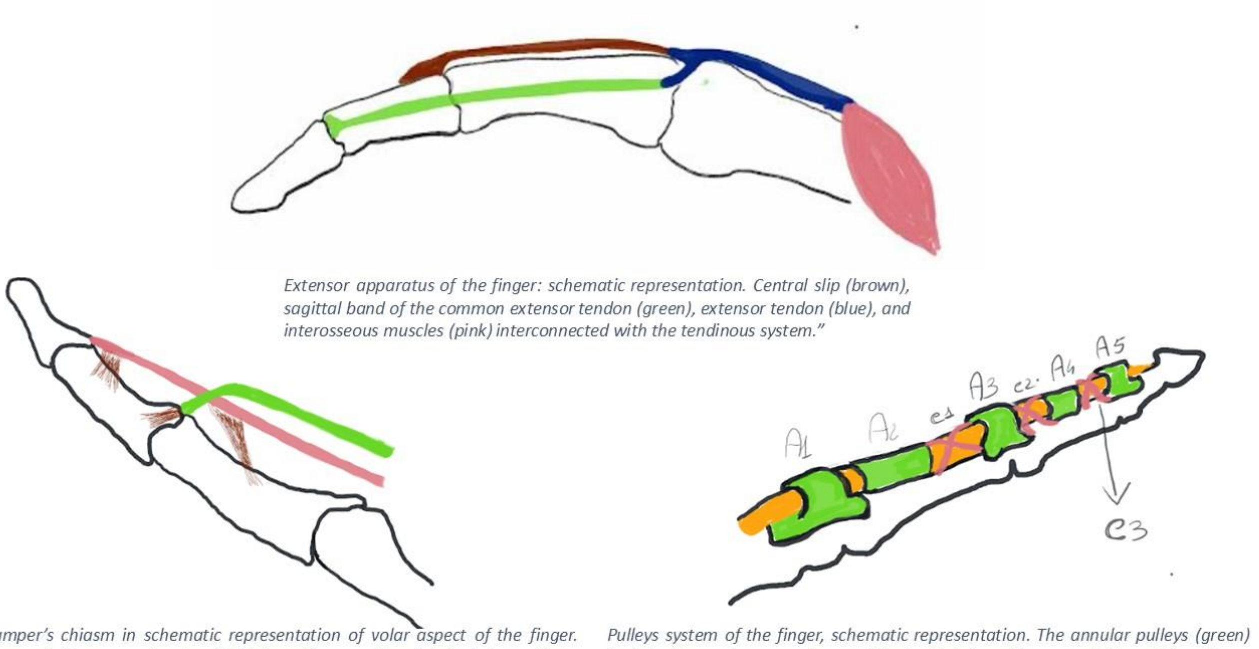

Extensor apparatus of the finger: schematic representation. Central slip (brown), sagittal band of the common extensor tendon (green), extensor tendon (blue), and interosseous muscles (pink) interconnected with the tendinous system."

Camper's chiasm in schematic representation of volar aspect of the finger. Flexor digitorum profundus (pink) inserting on the distal phalanx, flexor digitorum superficialis (green) inserting on the proximal phalanx, and vincula (red) supplying the flexor tendons."

Pulleys system of the finger, schematic representation. The annular pulleys (green) include A1, most commonly associated with trigger finger, and A3, positioned over the volar plate. The cruciate pulleys (pink) are interposed between the annular pulleys, while the flexor tendon system is shown in orange

Figure 1 Schematic representation of the digital anatomy relevant to dactylitis, including flexor and extensor tendons, tendon sheaths, pulleys, and joint structures, which represent key sites of inflammation in psoriatic arthritis

Dorsal region

The extensor apparatus of finger is a complex functional unit involving the extensor digitorum, lumbrical, and interosseous muscles. A key structure is the extensor hood, which stabilizes the extensor tendon located over the dorsal surface of the proximal phalanx. It includes sagittal bands, oblique and transverse fibers, with sagittal bands playing a major role in maintaining tendon alignment and being frequently involved in inflammatory conditions. [10,12]Distal to the metacarpophalangeal joint, the extensor digitorum communis divides into three parts. The central slip inserts at the base of the middle phalanx, while the lateral bands run along each side of the proximal interphalangeal joint.[10]All bony insertions of the central slip display fibrocartilage. These structures form part of the synovio-entheseal complex, which has been implicated in the pathogenesis of PsA[13,14]

Volar region

The volar region of the finger houses two flexor tendons: the flexor digitorum superficialis (FDS) and the flexor digitorum profundus (FDP). These tendons are enclosed within a synovial sheath that extends from the neck of the metacarpal to the distal interphalangeal joint. [15]The fibrous sheath stabilizes tendon position.[16] The intersection of these tendons forms the Camper's chiasm. [12]The flexor tendons are supplied by the digital arteries through a complex vascular network supported by connective tissue strands, known as vincula. [17]Also located in the volar region, the volar plates are fibrocartilaginous structures that reinforce the stability of the joints. Each finger (except the thumb) typically also contains five annular pulleys (A1–A5) and three cruciform pulleys (C1–C3).[18] Their function is preventing tendon bow-stringing during the digit flexion.[12,19]

In PsA, pulley are relevant sites of inflammation, supporting their role as functional entheses.[16] Notably, in PsA, fibrocartilage of the A2 and A4 pulleys can develop enthesophytes—bony projections at the enthesis—which may represent signs of inflammatory pathology.[20]

Lateral structures

Collateral ligaments are the main lateral stabilizers of the digital joints. They are classified as proper collateral ligaments, (limiting ulnar and radial deviation) and accessory. They insert into bone and the volar plate, and chronic microtrauma or inflammation can lead to their structural damage.[10]

Nail involvement is likely to be encountered in the context of dactylitis, particularly in PsA, where it represents a very common feature. In this setting, the nail should not be regarded merely as a cutaneous appendage, but rather as a structure closely connected to the distal phalanx in the so-called nail–enthesis complex. Anatomically, nail consists of a fibrous laminar structure, the nail plate, which overlies the nail matrix and originates from the nail bed. The nail bed extends deeply to form the nail root, a highly vascularized region that is in continuity with both the distal interphalangeal joint capsule and the extensor tendon. Between the nail plate and the nail bed lies a rich neurovascular network embedded within connective tissue. This close anatomical relationship supports the link between nail and joint inflammation in PsA. Inflammation at the extensor tendon enthesis has also been demonstrated by MRI.[21] Nail changes may therefore reflect the involvement of adjacent structures—including the joint, enthesis, and tendons—which are also implicated in dactylitis. Accordingly, the coexistence of nail involvement and diffuse digit inflammation may help clinicians suspect PsA.[22]

Reviewer #1, Comment 4: "*How much does the 'digital polyenthesitis' concept play a role in defining inflammatory and mechanical dactylitis? I would suggest that the authors focus on the mechanistic core of dactylitis and polyenthesitis.*"

Response: Thank you for this insightful comment. We have revised the manuscript to better clarify the role of the "digital polyenthesitis" concept in the pathogenesis of dactylitis. In particular, we have removed the detailed anatomical description of the entheses involved and have moderated the emphasis on digital polyenthesitis as a unifying mechanism. Current evidence suggests that dactylitis is a heterogeneous condition, resulting from the interplay of multiple structures.

Revised Text:

In 2015, Tan et al. described inflammation of small entheses associated with tendon pulleys—termed functional entheses—which are subject to repetitive mechanical stress. This led to the concept of "digital polyenthesitis" as a mechanism underlying dactylitis in PsA.[80] *However, the digital polyenthesitis model does not fully explain all forms of dactylitis; current evidence suggests that dactylitis pathophysiology is heterogeneous, involving both entheseal and non-entheseal structures, with varying contributions depending on the stage of the disease.*[80]

Reviewer #1, Comment 5: **"***What differentiates inflammatory and non-inflammatory dactylitis mechanistically? A bit of detail on the shared and divergent mechanistic theories across these etiologies will be helpful"*

Response: Thank you for this insightful comment. We have now expanded the manuscript to clarify the mechanistic distinction between inflammatory and non-inflammatory dactylitis. In particular, we emphasize the drivers underlying inflammatory forms (that is, the involvement of different cytokine mediators of inflammation through aberrant activation of the immune system) compared with non-inflammatory conditions (vascular mechanisms, microbial colonization, or abnormal soft tissue proliferation), which nonetheless lead to the same clinical phenotype—specifically, swelling of the entire digit, clinically identified as dactylitis.

Revised Text:

Etiopathogenetic considerations in dactylitis

Clinically, dactylitis presents as swelling of the entire digit. However, the underlying etiopathogenetic mechanisms may differ (inflammatory vs. non-inflammatory), as may the specific anatomical structures involved, which are not necessarily all affected in every case.

A well-established model of inflammatory dactylitis is that seen in PsA, where a multicompartimental process involves flexor tenosynovitis, soft tissue edema, synovitis, and enthesitis of pulleys and other structures in genetically predisposed individuals. An initial biomechanical insult, may trigger an aberrant innate immune response with increased production of pro-inflammatory cytokines.[25]

In other forms, although a similar diffuse swelling of the digit is observed, there is no primary immune-mediated inflammation. In infectious forms, the primum movens is microbial invasion, while in sickle cell disease it is an ischemic/vaso-occlusive process; in both cases, the inflammatory response represents a secondary amplification.[26,27]

Similarly, in mechanically induced forms such as pachydermodactyly, there are shared features with inflammatory dactylitis, including the role of mechanical stress as a trigger. However, while in inflammatory dactylitis mechanical stress promotes cytokine release, in pachydermodactyly induces fibroblastic proliferation without significant immune involvement.[28]

Reviewer #1, Comment 6: **"***Flexor tenosynovitis is described both in the etiopathogenesis and imaging sections".*

Response: thank you for this comment. We have revised the manuscript accordingly by removing the description of flexor tenosynovitis from the etiopathogenesis section of PsA, as it was already extensively covered in the imaging (ultrasound) section and therefore appeared redundant.

Reviewer #1, Comment 7: **"***The table on 'summary of dactylitis not related to rheumatologic disorders' is excellent and gives ample room to concise the text around that. It would be great if the authors could manage some clinical images of non-inflammatory dactylitis"*

Response: thank you for your positive feedback on the table. In response to your suggestion, we have added clinical images illustrating forms of dactylitis not only non-inflammatory but also not related to rheumatologic disorders, including both infectious and non-infectious etiologies

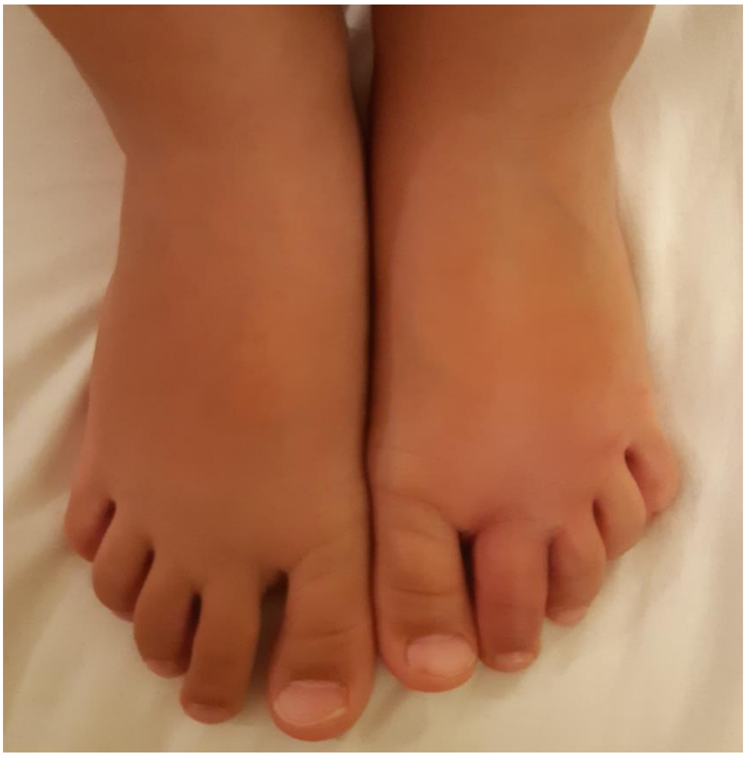

Figure 2

Blistering distal dactylitis in a 5-year-old girl presenting with acute swelling of the second toe of the left foot following an insect bite. The patient had no personal or family history of psoriasis. Approximately 24 hours after the insect bite, she developed fever and local erythema, consistent with an infectious dactylitis likely due to bacterial entry through a minor skin lesion. Complete resolution was achieved after antibiotic therapy.

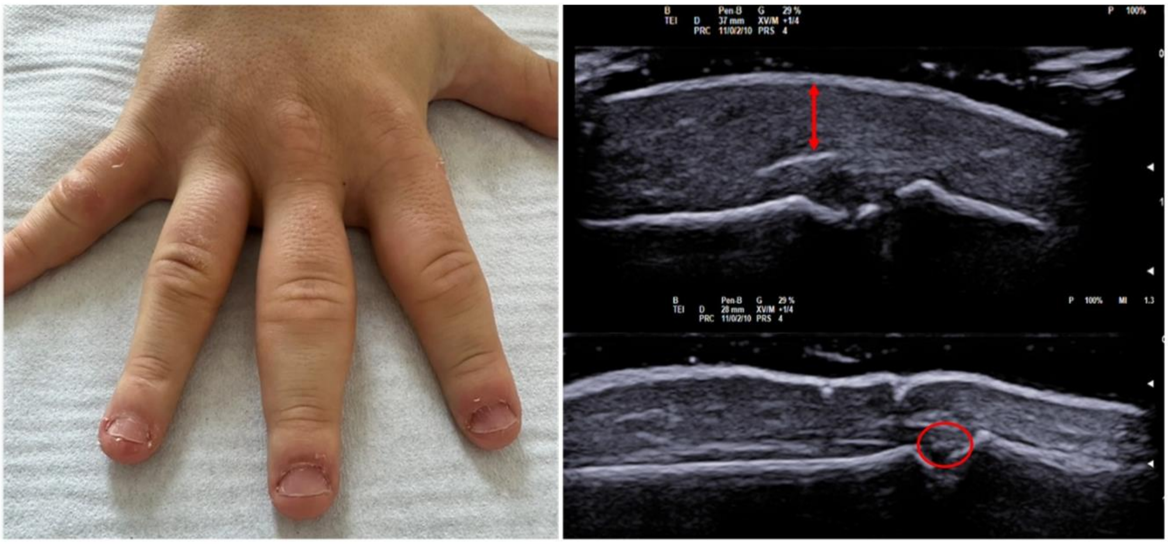

Figure 3 Dactylitis in pachydermodactyly. Left: Chronic, painless swelling of the third finger in an adolescent male. Right: Ultrasound images showing subcutaneous soft tissue thickening (arrow) and, on palmar longitudinal scan, absence of joint capsule distension or extensor tendon inflammation (circle).

Reviewer #1, Comment 8. *"Dactylitis in the pediatric age group can be concise. It is information-overloaded in its current form."*

Response: We thank the Reviewer for this valuable observation. In accordance with this comment, we have substantially shortened and refocused the pediatric section, retaining only the elements most relevant to the aims of the review. In particular, we clarified that, in Blau syndrome (BS) and early-onset sarcoidosis (EOS)**,** digital involvement should not be interpreted as persistent classical dactylitis; rather, the same granulomatous process that in adults may manifest as dactylitis more often evolves over time into camptodactyly, a fixed fibrotic contracture reflecting chronic structural remodeling. We also condensed the discussion of clinical, histologic, and imaging findings to improve readability while preserving the key message.

Revised text:

Dactylitic involvement in pediatric forms of sarcoidosis is described in Blau syndrome (BS) and early-onset sarcoidosis (EOS).[60] In these disorders, caused by NOD2 mutations, the same granulomatous process that in adults produces dactylitis instead leads over time to camptodactyly, a fixed and fibrotic contracture of the digits.[61] Dactylitis reflects acute, reversible inflammatory activity, whereas camptodactyly testifies to chronic, structural remodeling and damage.[60,61]

Blau syndrome was first described in 1985, and together with EOS forms the inherited and sporadic manifestations of the same disorder. The classic triad is granulomatous arthritis, dermatitis, and uveitis, typically arising in the first years of life. Over time, the persistence of granulomatous inflammation remodels periarticular tissues, inducing capsular fibrosis and tendon shortening that culminate in camptodactyly. Unlike sarcoid dactylitis, which is painful, swollen, and reversible, Blau/EOS camptodactyly is painless, fixed, and permanent.[62–64] Histology reveals non-caseating granulomas with epithelioid histiocytes, multinucleated giant cells, and dense lymphocytic coronas. Immunohistochemistry demonstrates prominent IFN-γ, IL-6, and IL-17 expression, consistent with pathogenic activation of both Th1 and Th17 pathways.[62,64] Clinically, BS /EOS camptodactyly is bilateral, symmetric, and predominantly affects the proximal interphalangeal joints. Unlike erosive

arthritides, joint surfaces remain preserved, and destructive changes are rarely observed on radiographs. In BS/EOS, ultrasound and MRI show synovial hypertrophy, Doppler-positive tenosynovitis, and fibrotic remodeling of periarticular tissues. The therapeutic approach to camptodactyly in BS/EOS is early and sustained suppression of granulomatous inflammation by corticosteroids and DMARDs to prevent irreversible contractures and other manifestations. Once established, camptodactyly is essentially refractory to systemic therapy, and orthopedic correction provides at best partial functional benefit.[62]

Reviewer #1, Comment 9: "*Imaging details can be grouped together. For instance, in the section on calcium hydroxyapatite deposition disease, the authors have mentioned imaging. This could be moved to the imaging section to maintain uniformity.*"

Response: Thank you for this helpful suggestion. We have revised the manuscript accordingly. Specifically, imaging details related to gout and calcium hydroxyapatite deposition disease have been removed from the clinical description and incorporated into the imaging section.

Revised Text:

Microcrystalline dactylitis *has been described in microcrystalline arthropaties.*
Gout is a form of inflammatory arthritis characterized by precipitation of sodium monourate (MSU) crystals in joint and soft tissues following chronic hyperuricemia. It has been reported in 5 to 9.6% of patients with gout.[48] *Dactylitis was observed in the polyarticular form and appeared to be a predictor of disease severity. In fact, patients with dactylitis exhibited a longer disease duration, greater joint involvement, higher serum uric acid levels, more tophi, higher ESR values and fulfilled more of the American College of Rheumatology/European League Against Rheumatism (ACR/EULAR) gout classification criteria beyond crystal identification.*[49,50] *Gout was confirmed at the time of presentation by the identification of MSU crystals, either aspirated from soft-tissue tophi of the affected digits or from the adjoining joints.*

Calcium hydroxyapatite deposition disease may lead to crystal deposition in tendons (referred to as calcific tendinitis), a common condition that may be asymptomatic or cause acute or chronic symptoms. Calcific tendinitis has been documented in nearly every tendon, with the shoulder being the most frequently affected site (approximately 60%), followed by the tendons of the hip, fingers, toes, and wrists.[51–54]*Acute calcific tendinitis affecting the fingers has been reported and should be considered a potential cause of hand dactylitis. Acute, self-limiting resorptive episodes typically present with sudden, intense pain and localized inflammation, with symptoms generally resolving within 4 to 7 days.*[55]

Reviewer #1, Comment 10: **"***Dactylitis in spondyloarthritis is generally mentioned as active and chronic, and not acute and chronic".*

Response: Thank you for pointing this out. We have carefully revised the manuscript and corrected all instances where "acute" dactylitis was incorrectly used instead of "active" dactylitis.

Reviewer #1, Comment 11: **"***I would describe clinical and imaging scoring systems separately. I would describe clinical and imaging scoring systems separately. The authors have clubbed LDI and DACTOS together as measurement tools, which could confuse the reader."*

Response: Thank you for this helpful comment. We have revised the manuscript accordingly by separating clinical and imaging scoring systems to improve clarity. Specifically, the clinical tool (LDI) has been included in the section on "clinical assessment of psoriatic arthritis", while the ultrasonographic scores (DACTOS and GLOUDAS) have been moved to the imaging section.

Revised Text:

Clinical assessment of psoriatic dactylitis

The development of new treatments for PsA has required the introduction of measurement tools to quantify dactylitis. The Leeds Dactylitis Index (LDI), proposed by Helliwell et al., is based on digital circumference and tenderness, comparing the affected digit with its contralateral counterpart (if unaffected). A ≥10% difference in circumference defines dactylitis. Reference tables are provided for cases of symmetric involvement of two digits. This method has shown good inter- and intra-observer reliability but may be limited in obese patients, where excess fatty tissue hampers measurement.[25,67]

Ultrasonographic assessment in PsA

Imaging-based tools have been developed to provide a more comprehensive evaluation of dactylitis. DACTOS score is currently the most widely adopted tool for evaluating involvement of the anatomical components in ultrasound dactylitis. It is sensitive to change and reliable in daily practice.[73]*The DACTylitis glObal Sonographic (DACTOS) score integrates ultrasonographic findings into a global score from 0 to 25. It has demonstrated high reliability among expert ultrasonographers, although subcutaneous edema and peritendinitis showed slightly lower concordance.*[74] *The OMERACT Global Ultrasound Score for Dactylitis (GLOUDAS), developed by Naredo et al., assesses the main anatomical structures involved in psoriatic dactylitis. Through Delphi consensus, 12 digital entheses were selected for the final version of the score. Intra-observer reliability was excellent.*[75] *Zhao et al. used DACTOS score to identify subclinical dactylitis on ultrasound, finding an association with a more severe PsA phenotype.*

Reviewer #1, Comment 12: **"***Could the authors include a reference for the statement on using 7.5-13.5 MHz probes for dactylitis assessment in the ultrasound section?"*

Response: thank you for this comment. We have now included appropriate references to support the statement on the use of 7.5–13.5 MHz probes. The use of high-frequency linear transducers was standardized by the EULAR task force in 2017 for the assessment of superficial structures, such as the small joints of the hands and the associated ligamentous and tendinous structures (Möller et al., 2017). In addition, the use of these probes has been supported by further studies demonstrating a correlation between specific ultrasound findings—particularly soft tissue edema and flexor tenosynovitis—and the inflammatory phases of dactylitis in psoriatic arthritis (Girolimetto et al., 2020,Felbo SK, et al. DOI: 10.1007/s10067-020-05483-9 and Dubash, S. et al. DOI: 10.1136/annrheumdis-2021-220964)

Reviewer #1, Comment 13: **"***Again, the ultrasound section is too long and includes a table".*

Response Thank you for this important comment. We have revised the imaging section by reducing the main text and removing redundant descriptions. In particular, detailed information has been

consolidated into the table. This approach reduces duplication and improves clarity without loss of information.

Revised Text:

Role of imaging in Dactylitis

Ultrasound

The advent of high-frequency probes in modern US systems has enabled highly detailed imaging of small and superficial anatomical structures, including the digits. For the accurate assessment of dactylitis, the use of 7.5–13.5 MHz probes is recommended; although higher frequencies (up to 22 MHz) can further improve resolution, particularly for visualizing the pulleys.[1,68]

On US, dactylitis typically presents as a combination of synovial and extra-synovial inflammatory changes, including joint synovitis, enthesitis, flexor tendon tenosynovitis, and subcutaneous tissue oedema (STO). All ultrasonographic abnormalities observed in dactylitis are defined according to OMERACT definitions. [46,69,70] *(Table 3 and Figure 5)*

Extracapsular structures, including the extensor hood, are key contributors to the pathogenesis of psoriatic arthritis. The extensor hood can be visualized as a thin echogenic band, with the characteristic "seagull sign" in transverse view. These structures, together with the central slip enthesis and adjacent synovium, form the synovio-entheseal complex (SEC). [12,46,71] *Further studies showed that extracapsular involvement correlated more strongly with symptomatic ("hot") dactylitis than with asymptomatic ("cold") dactylitis, the latter more frequently presenting with synovitis.*[72] *In early, untreated PsA, it has been observed that hot dactylitis was most frequent in toes (particularly the second and fourth).*[66]

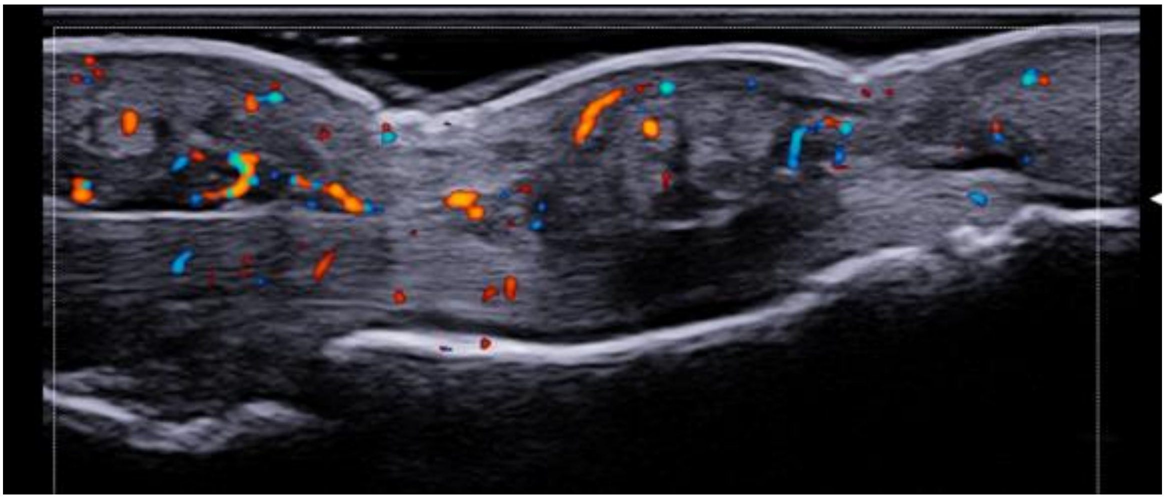

Figure 5 Ultrasound features of active dactylitis. Grey-scale longitudinal reconstruction of the volar aspect of a finger affected by active dactylitis, obtained through multiple scans, showing thickened, iso-hypoechoic subcutaneous oedema and distension of the flexor tendon sheath surrounding the tendon. Power Doppler imaging demonstrates a scalloped appearance of the flexor tendon in regions lacking pulley support, with marked intra-tendinous hypervascularisation. (PD: power Doppler).

Ultrasonographic assessment in PsA

Imaging-based tools have been developed to provide a more comprehensive evaluation of dactylitis. DACTOS score is currently the most widely adopted tool for evaluating involvement of the anatomical components in ultrasound dactylitis. It is sensitive to change and reliable in daily practice.[73]The DACTylitis glObal Sonographic (DACTOS) score integrates ultrasonographic findings into a global score from 0 to 25. It has demonstrated high reliability among expert ultrasonographers, although subcutaneous edema and peritendinitis showed slightly lower concordance.[74] The OMERACT Global Ultrasound Score for Dactylitis (GLOUDAS), developed by Naredo et al., assesses the main anatomical structures involved in psoriatic dactylitis. Through Delphi consensus, 12 digital entheses were selected for the final version of the score. Intra-observer reliability was excellent.[75] Zhao et al. used DACTOS score to identify subclinical dactylitis on ultrasound, finding an association with a more severe PsA phenotype.

Anatomical Structure	***Normal Ultrasound Appearance***	***Pathological Findings in Dactylitis***
Synovium	*Hypoechoic thin layer within capsule; no PD signal*	*Thickened hypoechoic tissue, non-displaceable, poorly compressible, with or without PD activity (synovitis)*
Flexor Tendon Sheath	*Thin, hyperechoic line surrounding tendon*	*Hypoechoic/anechoic halo, sheath distension ('festooned appearance'), ± PD signal (tenosynovitis)*
Extensor Tendon	*Fibrillar echotexture, centrally located under extensor hood*	*Altered echogenicity, displacement outside hood (sagittal band rupture), increased PD signal*
Extensor Hood & Sagittal Bands	*Thin echogenic band; transverse 'seagull' sign*	*Thickening, discontinuity, PD signal; subluxation of tendon*
Central Slip & Enthesis	*Continuous fibrillar structure inserting on middle phalanx*	*Enthesitis with hypoechogenicity, thickening, PD signal; cortical irregularity or avulsion*
Peritendinous Soft Tissues (Extensor)	*Uniform echotexture, no PD activity*	*Hypoechoic swelling surrounding tendon (PTI), ± PD signal*
Subcutaneous Tissues	*Normal echogenic subcutaneous fat*	*Diffuse swelling (STO), increased PD signal from finger pad to MCP joint*
Pulleys (A1–A5, C1–C3)	*Linear anisotropic band ≤0.60 mm; hyperechoic volar portion*	*Increased thickness, hypoechogenicity, partial or complete rupture; bowstring deformity (A2/A4), trigger finger (A1)*
Collateral Ligaments	*Hyperechoic fibrillar bands at lateral joint margins*	*Thickening, partial rupture, altered echotexture, instability on dynamic scan*

Table 1 Ultrasound Assessment of Dactylitis

Magnetic resonance imaging

MRI offers unparalleled visualization of soft tissues, joints, and entheses, providing diagnostic insights not achievable with other imaging modalities. Gadolinium-based intravenous contrast is routinely used to enhance the detection of abnormalities and to distinguish effusion from synovitis.

A recommended MRI approch for dactylitis includes T1-weighted, T2-weighted, and short tau inversion recovery (STIR) sequences.[76] Bone marrow oedema appears as poorly defined subcortical hyperintensity on fluid-sensitive sequences and is often accompanied by extensive soft tissue oedema with a "honeycomb" pattern on T2-weighted images.[19,77,78]

Previous MRI studies in PsA have shown that dactylitis is primarily associated with flexor tendon tenosynovitis, while joint effusion is typically less prominent.[79] In 2015, Tan et al. described inflammation of small entheses associated with tendon pulleys—termed functional entheses—which are subject to repetitive mechanical stress. This led to the concept of "digital polyenthesitis" as a

mechanism underlying dactylitis in PsA.[80] *However, the digital polyenthesitis model does not fully explain all forms of dactylitis; current evidence suggests that dactylitis pathophysiology is heterogeneous, involving both entheseal and non-entheseal structures, with varying contributions depending on the stage of the disease.*[80]

MRI can also demonstrate similar patterns of tenosynovitis in traumatic conditions, interpreted as a Koebner-like phenomenon.[81]

MRI can also contribute to assessment. The Psoriatic Arthritis Magnetic Resonance Imaging Hand Scoring System (PsAMRIS-H), developed by OMERACT, can identify features of dactylitis and evaluate treatment response with high intra- and inter-reader reliability.[45,82] *Although MRI is highly accurate in detecting tenosynovitis, ultrasound is useful for identifying insertional enthesitis and has been used to visualize enthesitis at the deep flexor tendon insertions in PsA.*[83,84]

Although most imaging data derive from PsA, imaging findings (US, X-ray, and MRI) may also be relevant in dactylitis of other etiologies, including microcrystalline diseases, where they can contribute to the differential diagnosis.

MRI findings showed that digit involvement in gouty dactylitis can result from different pathological mechanisms: a true inflammatory condition (characterized by arthritis, tenosynovitis, and soft tissue edema) as well as a non-inflammatory process involving accumulation of hyperintense material circumferential (possibly MSU) in the subcutaneous tissue of the affected digit, known as dactylopathy.[48,85]

US and MRI may show acute synovitis of the flexor sheath in microcrystalline diseases.[56] *On MRI, periarticular calcifications appear as low-signal-intensity deposits on both T1- and T2-weighted images, and may be overlooked. MRI findings of surrounding soft-tissue and bone edema can lead to misdiagnosis as a more aggressive pathology, especially if MRI is the initial imaging modality used.*[52]

Reviewer #1, Comment 14: *"The authors claim that MRI assessment can be challenging due to the complex anatomy of the structures involved. The concept of polyenthesitis was based on a high-resolution MRI study. So, this sentence doesn't make sense."*

Response: thank you for this important comment. We agree that our previous statement was unclear and potentially misleading. Our intention was not to question the ability of MRI to depict the anatomical complexity of dactylitis, but rather to refer to its limited accessibility and the level of expertise required for proper interpretation, particularly in non-specialized settings. To avoid any misunderstanding, we have removed this statement from the revised manuscript.

Reviewer #1, Comment 15: *"In the MRI section, what do the authors mean by this sentence? "The examination typically begins with whole-hand imaging to detect all abnormalities at the highest available spatial resolution, followed by targeted imaging of the affected digit with a reduced field of view." This can only confuse the reader."*

Response: Thank you for this comment. We agree that the sentence was not sufficiently clear and may be confusing for the reader. Our intention was to describe a commonly adopted MRI acquisition approach, followed by a more focused high-resolution assessment of the affected digit. To improve clarity, we have removed it from the revised manuscript.

Reviewer #1, Comment 16: *"The authors have recommended an MRI protocol for dactylitis. What's the reference for this? Also, what's the reference to mention that the ultrasound is more sensitive than MRI for dactylitis imaging?".*

Response: thank you for this comment. We have now included an appropriate reference to support the proposed MRI approach for the assessment of dactylitis. We apologize for the inappropriate use of the term "protocol," as the cited study describes a comprehensive imaging approach rather than a standardized protocol. [Scarano E, et al. Morphologic, dynamic and high-resolution microscopy MRI in early-onset spondyloarthritis finger dactylitis. Skeletal Radiol. 2023 Jun; DOI: 10.1007/s00256-022-04218-y]

Regarding the comparison between imaging modalities, we acknowledge that the statement suggesting that ultrasound may be more sensitive than MRI was partially incorrect, as our intention was to present these techniques as complementary rather than hierarchical. In fact, ultrasound offers distinct advantages, including excellent accessibility and real-time assessment, but also has limitations, such as poorer visualization of deep structures and the inability to detect bone marrow edema [Bakewell CJ, et al., OMERACT Ultrasound Task Force. Ultrasound and magnetic resonance imaging in the evaluation of psoriatic dactylitis: status and perspectives. J Rheumatol. 2013; DOI: 10.3899/jrheum.130643].

Minor comments:

Reviewer #1, Comment 1***:*** *There are some minor grammatical errors in the sentences that need to be addressed.*

Response: Thank you for this comment. We have carefully revised the manuscript to correct minor grammatical errors**.**

Reviewer # 2(Remarks to the Author): **"***The manuscript reviews many aspects of dactylitis as a symptom of diverse medical conditions. It is a Well-written and interesting paper. I have several minor suggestions for the authors*"

Response: Thank you for your comment. We have carefully revised the manuscript and incorporated the minor suggestions as you indicated.

Reviewer #2, Comment 1: *"line 76. Please, re-check the grammar"*

Response: Thank you for your comment. The sentence "*Dactylitis (from the Greek word "dactylos", meaning digit) is a medical condition commonly known as "sausage digit*" has been rephrased as follows: "Dactylitis (from the Greek word 'dactylos', meaning digit) is a medical condition commonly referred to as 'sausage digit', a term describing the diffuse swelling of a finger or toe."

Reviewer #2, Comment 2: **"***line 242. The phrase 'bone expansion due to destruction' is unclear. Please, explain"*

Response: Thank you for your comment. We have clarified this point in the revised manuscript. In this context, the term "expansion" refers to the combination of trabecular bone rarefaction and periosteal thickening, which together contribute to the typical fusiform appearance of the digit.

Reviewer #2, Comment 3: **"***line 247. Dactylitis due to non-tuberculous mycobacteria, particularly M. marinum, is not uncommon at all and should be discussed in more depth, better in a separate sub-chapter, including epidemiology and tips for diagnosis"*

Response: Thank you for your comment. We have revised the text accordingly and expanded this section to better address dactylitis associated with non-tuberculous mycobacteria, particularly *Mycobacterium marinum*. The original sentence: "*Atypical mycobacteria linked to saltwater exposure, particularly Mycobacterium marinum, can lead to cellulitis that may progress to dactylitis if untreated. The diagnosis is often delayed because of the long incubation period (around one month), making biopsy critical for confirmation*" has been reformulated into a brief paragraph describing the clinical presentation, epidemiological context, and diagnostic challenges of *M. marinum*-related dactylitis.

Revised text:

Non-tuberculous mycobacterial dactylitis. *Atypical mycobacteria linked to saltwater exposure, (including domestic fish tanks), particularly Mycobacterium marinum, can lead to cellulitis that may progress to dactylitis if untreated. The soft tissue infection has an incidence of 0.27 cases per 100,000 individuals in the United States.*[30] *M. marinum penetrates the skin through direct injury; the incubation period ranges between 3 weeks and 9 months. Clinical presentations of Mycobacterium marinum infection are commonly observed in immunocompetent patients as single or limited cutaneous lesions, typically appearing as ulcerated nodules, and may also involve arthritis and tenosynovitis. In some cases, disseminated infections can occur, associated with pulmonary involvement and other systemic manifestations. Diagnosis is often delayed due to the long incubation period, making biopsy critical for confirmation. M. marinum is a naturally multidrug-resistant organism. Antibiotic therapy with clarithromycin, trimethoprim, and ciprofloxacin is considered effective, while combination therapy is required in cases of deeper infections.*[31,32]

Reviewer #2, Comment 4: "*Please, add when to suspect and how to diagnose GCTTS"*

Response: Thank you for your comment. We have revised the section on Giant Cell Tumour of the Tendon Sheath by including additional details on this condition should be suspected and how it can be accurately diagnosed.

Revised text:

Giant Cell Tumour of the Tendon Sheath (GCTTS) *is one of the most common tumors of the hand in adults. It arises from the proliferation of synovial cells in tendon sheaths, producing elastic nodules intimately connected to the tendon. It typically occurs in middle-aged women and is generally slow-growing. This condition should be suspected when a painless mass appears in the volar region of the digit. In addition to physical examination, imaging techniques such as plain radiographs or MRI can aid in the differential diagnosis. Indeed, in the early stages, these nodules may displace adjacent structures and mimic the appearance of dactylitis.*[12,41]

Reviewer #2, Comment 5: "*line 347. Non-tuberculous mycobacterial dactylitis should be added to the table"*

Response: Thank you for your comment. We have added a new row to the table addressing non-tuberculous mycobacterial dactylitis.

Reviewer #2, Comment 6: "*lines 115, 120, 354, 423 refer to the prevalence of PsA-dactylitis. Why is it different every time?"*

Response: Thank you for your comment. The differences in the reported prevalence of PsA-related dactylitis reflect variability across studies, which may depend on patient populations, disease stage, and study design. We have clarified this point for each prevalence value reported throughout the manuscript.

Revised text: *"Epidemiological data vary across studies and are influenced by differences in patient populations,"*

Reviewer #2, Comment 7: *"line 367. Should 'higher' be used in place of 'elevated'?"*

Response: Thank you for your suggestion. We have replaced "elevated" with "higher" where appropriate.

Reviewer #2, Comment 8: **"***line 367. ACR criteria for what?"*

Response: Thank you for your comment. We have revised the text to clarify that we are referring to the American College of Rheumatology/European League Against Rheumatism gout classification criteria, and we have included the appropriate reference.

Revised text:

"In fact, patients with dactylitis exhibited a longer disease duration, greater joint involvement, higher serum uric acid levels, more tophi, higher ESR values and fulfilled more of the American College of Rheumatology/European League Against Rheumatism (ACR/EULAR) gout classification criteria beyond crystal identification." [Neogi T, et al. Gout classification criteria: an American College of Rheumatology/European League Against Rheumatism collaborative initiative. Ann Rheum Dis. 2015 Oct; [DOI: 10.1136/annrheumdis-2015-208237].

Reviewer #2, Comment 9: **"***line 369. Please change to 'aspirated from soft-tissue tophi"*

Response: Thank you for your comment. We have revised the text as you suggested.

Revised text: *Gout was confirmed at the time of presentation by the identification of MSU crystals, either aspirated from soft-tissue tophi of the affected digits or from the adjoining joints.*

Reviewer #2, Comment 10: **"***lines 120 and 391 - again, the mentioned prevalence of sarcoid dactylitis is different"*

Response*:* Thank you for your comment. The differences in the reported prevalence of sarcoid dactylitis likely reflect variability across studies, which is influenced by differences in patient populations—particularly ethnic background—as well as by the type of case series analyzed. We have clarified this point in the revised manuscript.

Revised text: *Epidemiological data that vary across studies and are influenced by differences in patient populations indicate prevalence rates of 39% in PsA, 17% in sarcoidosis, 9.5–12% in other SpAs, and 5–9.6% in gout.*[6]

Reviewer #2, Comment 11: *lines 424-426. Please, re-check the grammar*

Response: Thank you for your comment. All grammatical corrections are shown using track changes.

Reviewer #2, Comment 12: "*lines 428. Please, discuss the point of view that dactylitis is a subtype of enthesitis or ligamentitis"*

Response: Thank you for your comment. Our intention to describe dactylitis as a subtype of enthesitis or ligamentitis is based on the etiopathogenetic model proposed by McGonagle et al., largely derived from experimental animal studies. According to this hypothesis, the primum movens of dactylitis is the initiation of inflammation at periarticular insertional sites, including tendons and ligaments. In particular, the digital flexor tendon pulleys—regarded as "functional entheses"—appear to play a pivotal role, along with other entheseal structures such as collateral ligaments and nail-associated anchorage complexes. These extra-articular components are key contributors to the inflammatory process in psoriatic arthritis and may, although currently unproven, act as the initial sites of inflammation, subsequently promoting articular involvement and ultimately resulting in diffuse inflammation of the entire digit.

Reviewer #2, Comment 13: **"***lines 437-440 probably should appear at line 351, after the citation 33"*

Response: Dear Reviewer, thank you for your suggestion. We sincerely apologize for the confusion caused by an inconsistency in the line numbering within the revised manuscript, which likely resulted from a formatting problem during the editing process. In response to your comment "*lines 437–440 probably should appear at line 351, after the citation 33"*, we have carefully re-examined the manuscript and understand that you were referring to the paragraph: "*According to its clinical features PsA dactylitis can be classified as acute (hot) or chronic (cold). The former is is characterized by swelling, pain, and, sometimes, redness, the latter by swelling without pain or other signs of inflammation. Recognition of dactylitis can facilitate early PsA diagnosis—within 12 months of symptom onset—and improved treatment.*[56,57] Accordingly, this paragraph has now been moved to line 338, as suggested, immediately after 'a hallmark of PsA,' because we have removed the paragraph referring to reference 33 to reduce the overall length of the review. We thank you again for your helpful comment. Should we have misunderstood your suggestion, we would be happy to revise the correction.

Revised text:

***Psoriatic dactylitis** is considered a hallmark of PsA. According to its clinical features PsA dactylitis can be classified as active (hot) or chronic (cold). The former is characterized by swelling, pain, and, sometimes, redness, the latter by swelling without pain or other signs of inflammation. Recognition of dactylitis can facilitate early PsA diagnosis—within 12 months of symptom onset—and improved treatment.*[43,44]

Reviewer # 3(Remarks to the Author), Comment 1: **"***A main component of every digit is the nail. It has been completely overlooked in the manuscript, both in the anatomy and various causes of dactylitis."*

Response: thank you for your comment. We agree that the nail is an important component of the digit. We have added this aspect within the anatomical section describing the components of dactylitis, in order to better contextualize it without losing the overall focus of the manuscript. In addition, in light of your third comment, we have included a brief reference to the differential diagnosis with sarcoidosis.

Revised text:

Nail involvement is likely to be encountered in the context of dactylitis, particularly in PsA, where it represents a very common feature. In this setting, the nail should not be regarded merely as a cutaneous appendage, but rather as a structure closely connected to the distal phalanx in the so-called nail–enthesis complex. Anatomically, nail consists of a fibrous laminar structure, the nail plate, which overlies the nail matrix and originates from the nail bed. The nail bed extends deeply to form the nail root, a highly vascularized region that is in continuity with both the distal interphalangeal joint capsule and the extensor tendon. Between the nail plate and the nail bed lies a rich neurovascular network embedded within connective tissue. This close anatomical relationship supports the link between nail and joint inflammation in PsA. Inflammation at the extensor tendon enthesis has also been demonstrated by MRI.[21] Nail changes may therefore reflect the involvement of adjacent structures—including the joint, enthesis, and tendons—which are also implicated in dactylitis. Accordingly, the coexistence of nail involvement and diffuse digit inflammation may help clinicians suspect PsA.[22]

The main nail changes in psoriasis may involve either the nail matrix or the nail bed. When the nail matrix is affected, the most common findings include pitting (small punctate depressions on the nail plate), leukonychia (white spots), and crumbling (nail plate fragility). When the nail bed is involved, subungual hyperkeratosis and detachment of the nail plate (onycholysis) can be observed.[23] These alterations not only reflect the involvement of specific components of the nail unit, but may also help guide clinicians in the differential diagnosis with other nail manifestations seen in dactylitis of causes other than PsA (for example, the different pattern of nail involvement observed in sarcoid dactylitis).[24]

Reviewer # 3, Comment 2: "*Another cause of dactylitis is coeliac disease combined with vit D deficiency. A 30-year-old Pakistani female patient with osteomalacia and coeliac disease presenting with dactylitis (Jawad et al. Osteomalacia and coeliac disease presenting as isolated dactylitis Annals of the Rheumatic Diseases, 1988; 47, 692-694). The apparent dactylitis and the radiological bone changes resolved after treatment with a gluten free diet and calciferol, as did the radiological bone features of osteomalacia."*

Response: thank you for highlighting this interesting additional cause of dactylitis. We have now included coeliac disease associated with vitamin D deficiency among the non-inflammatory causes of dactylitis in the revised manuscript, and have added the corresponding reference.

Revised text:

***Dactylitis associated with coeliac disease and vitamin D deficiency** has also been reported. A 30-year-old Pakistani female patient with osteomalacia and coeliac disease presenting with dactylitis. The apparent dactylitis and the radiological bone changes resolved after treatment with a gluten free diet and calciferol, as did the radiological bone features of osteomalacia. [Jawad et al. Osteomalacia and coeliac disease presenting as isolated dactylitis Annals of the Rheumatic Diseases, 1988; 47, 692-694].*

Reviewer # 3, Comment 3: *"Sarcoid dactylitis is often associated with dystrophic nail changes. The nail in the affected finger appears brittle, thin, clubbed with subungual keratosis, longitudinal ridging, paronychia and brown discolouration of the nail bed. (Jawad AS. Sarcoid dactylitis with nail involvement. JRCPE 2022). https://doi.org/10.1177/14782715221113050.The nail changes need to be compared to the nail changes in psoriasis. The dactylitis in sarcoid can be restricted to part of the finger overlying the affected phalanx or phalanges. A plain X-ray is needed for the*

affected digit and the whole hand to look for the diagnostic bone changes of sarcoidosis in the phalanges."

Response: thank you for this helpful comment. We have expanded the description of nail involvement in sarcoid dactylitis within the section dedicated to sarcoidosis, including the clinical features you highlighted and the relevant reference.

Revised text:

Sarcoid dactylitis is often associated with dystrophic nail changes. The nail in the affected finger appears brittle, thin, clubbed with subungual keratosis, longitudinal ridging, paronychia and brown discolouration of the nail bed. The dactylitis in sarcoid can be restricted to part of the finger overlying the affected phalanx or phalanges. A plain X-ray is needed for the affected digit and the whole hand to look for the diagnostic bone changes of sarcoidosis in the phalanges. [Jawad AS. Sarcoid dactylitis with nail involvement. JRCPE 2022). https://doi.org/10.1177/14782715221113050]

Reviewer # 3, Comment 4: *"TB dactylitis. The onset is often gradual in comparison to BDD. We have seen 2 patients presenting with TB dactylitis while on anti TNF therapy for Crohn's disease in spite of negative gamma interferon test and a normal chest X-ray prior to starting therapy. The reader needs to be alerted to this observation."*

Response: thank you for this important comment. We have added a note in the section on tuberculous dactylitis to highlight its insidious onset and to alert readers to the possibility of its occurrence in patients receiving anti-TNF therapy despite negative baseline screening, as you suggested.

Revised text:

Tuberculous dactylitis *is an extremely rare localization of Mycobacterium tuberculosis, accounting for only 1%–3% of all tuberculosis cases. It occurs most frequently in children, typically affecting the phalanges of the hands, while multifocal involvement is exceptionally uncommon. Clinically, it presents with swelling and pain. Although rare, adult cases have also been reported, sometimes occurring in the absence of prior pulmonary symptoms. Notably, tuberculous dactylitis may also develop in patients receiving anti-TNF therapy despite negative baseline interferon-gamma release assays and normal chest radiographs. Radiographically, soft tissue swelling is observed, along with cystic cavities containing internal septations and cortical bone lytic lesions. Histological examination demonstrates caseous granulomas with epithelioid cells and Langhans-type giant cells. The characteristic "spina ventosa" appearance (resembling a "sail full of wind") results from trabecular bone rarefaction and periosteal thickening. Management requires appropriate anti-tubercular pharmacotherapy.*[7,29]

Reviewer # 3, Comment 5: *"If there are features specific to the type of dactylitis such as the nail changes in sarcoid and psoriatic dactylitis, they need to be described".*

Response: Thank you for this comment. As suggested, we have included a description of nail changes associated with different types of dactylitis (in particular, psoriatic and sarcoid dactylitis) to highlight their diagnostic relevance. This has been addressed in the revised manuscript (see first and third comments).

Reviewer # 3, Comment 6: *"A table comparing the clinical and imaging findings of dactylits in common condition would be useful to the reader"*

Response: Thank you for this valuable suggestion, which we greatly appreciated. We have now included tables comparing the main clinical and imaging features across the different etiologies of dactylitis, structured according to the proposed etiopathogenetic classification. Specifically, we have revised the original table by splitting it into two separate tables: one dedicated to dactylitis associated with rheumatologic disorders and the other to conditions not related to rheumatologic diseases. Imaging features have been incorporated into both tables. The revised manuscript now includes these new tables.

Revised Text:

**Category**	**Condition**	**Incidence / Typical Age**	**Clinical Features**	**Imaging findings**
**Non-inflammatory**	Sickle Cell dactylitis (Hand–Foot Syndrome)	Children aged 6 months–2 years; more frequent in colder months	Painful swelling of hands/feet; vaso-occlusive crises	On X-ray: lytic lesions, bone necrosis; osteosclerosis
	Pachydermodactyly	Adolescents; males > females	Symmetrical, painless, elastic swelling of PIP joints; absence of synovitis	Soft tissue thickening only on US
	Coeliac disease	Rare	Painless finger swelling	Radiological bone features of osteomalacia
**Neoplastic**	Osteoid Osteoma	Benign bone tumor, common in children	Localized pain/swelling;	Radiolucent nidus on X-Ray
	Giant Cell Tumor of the Tendon Sheath	Most common benign tumor of the adult hand	Firm, elastic nodules adherent to tendons, Painless mass of soft tissue	Key findings on magnetic resonance, particularly on T2-weighted sequences, include 'blooming' on gradient-echo images due to hemosiderin deposition
	Paraneoplastic Dactylitis	Rare; associated with solid tumors (e.g., ovarian cancer)	Recurrent swelling as a paraneoplastic manifestation	
**Infectious**	Tuberculous Dactylitis (Spina ventosa)	1–3% of skeletal TB; children > adults	Gradual onset; Painful swelling of hands/feets; may occur without pulmonary symptoms	Soft tissue swelling (US);<br>On X-ray: cystic cavities, lytic lesions, bone necrosis
	Non-tuberculous mycobacterial (M. marinum)	Associated with water exposure; incidence of 0.27 cases per 100,000 individuals	Cutaneous nodules/ulcers; may progress to tenosynovitis/arthritis; often after trauma	Severe tenosynovitis, soft tissue edema, but findings are often non specific
	Congenital Syphilis	~2% of skeletal manifestations of syphilis	Bilateral, symmetrical involvement;	Periosteal reaction; minimal soft tissue swelling
	Yaws (Treponema pertenue)	Rare; endemic areas	Phalangeal lesions similar to syphilis	Findings similar to syphilis
	Blistering Distal Dactylitis (BDD)	Children	Volar (occasionally dorsal) vesicles; rapid onset; non-painful, non-pruritic	Fluid collection (anechoic on US) in the soft tissues without articular involvement
	Leprosy (Hansen's Disease)	13% prevalence in large series (n ≈ 25,000)	Symmetrical joint involvement Tenosynovitis, nodules, panniculitis, erythema nodosum leprosum	On US soft tissue edema, tenosynovitis (non-specific),<br>On X- ray lytic lesions, bone erosion
	Lyme Borreliosis	Rare	Chronic atrophic acrodermatitis; nodules mimicking dactylitis, Swelling and pain	On US soft tissue edema, tenosynovitis and synovitis (non-specific)

**Parasitic**	Cutaneous Leishmaniasis	Extremely rare; 13 reported cases	Swelling, pain, granulomas with amastigotes; possible paronychia; exposure history	soft tissue swelling on US radiography may show lytic lesions or periosteal reaction

Table 2 Summary of dactylitis not related to rheumatologic disorders

**Condition**	**Prevalence / Typical Age**	**Clinical Features**	**Imaging findings**
Psoriatic dactylitis	Between 33 an 55%, but epidemiological data vary across studies	Painful or painless swelling; frequently asymmetric	Tenosynovitis, synovitis and soft tissue edema; enthesitis with hypoechogenicity, thickening and Power Doppler signal of extennsor tendon
Gout	About 5–9.6%	Polyarthritis; presence of tophi	severe soft tissue swelling, tenosynovitis, and tophi (hyperechoic masses on US)
Calcium hydroxyapatite deposition	Rare, rare case report	Acute pain due to calcific tendinitis; self-limiting	On US, hyperechoic deposits in extra-articular structures (tendons) and periarticular calcifications
Sarcoid dactylitis	Rare, epidemiological data vary across studies	May be asymptomatic; nail changes	On X-ray lytic lesions; honeycomb pattern
Blau syndrome / EOS	Rare, case reports	Camptodactyly; chronic, fixed, painless	Synovial hypertrophy
Calcium pyrophosphate deposition disease dactylitis	Rare, case reports	acute, diffuse finger swelling and functional impairment; achieved rapid remission following a short course of colchicine;	On US, synovial hypertrophy; severe soft tissue swelling, tenosynovitis

Table 3 Summary of dactylitis related to rheumatologic disorders

Reviewer # 3, Comment 7: "*Leishmaniasis: The epidemiology of this vector-born disease need to be included. Eastern hemisphere leishmaniasis refers to Leishmaniasis found in Africa, Europe, Asia, and Oceania (previously referred to Old World leishmaniasis). Western hemisphere leishmaniasis refers to the disease found in the Americas (New World leishmaniasis). Travel history to endemic areas needs to be emphasized. The onset of dactylitis in leishmaniasis is gradual and is characterised by excessive scarring of the skin of the digit. Nail changes are common and often the nail looks ischemic and it may fafl.*"

Response: Thank you for this comment. We have revised the section on leishmaniasis to incorporate the epidemiological aspects you highlighted, including the distinction between Old and New World leishmaniasis. We have also expanded the clinical description to emphasize the onset of dactylitis and the characteristic nail changes. The relevant reference has been added.

Revised text:

The epidemiology of this vector-borne disease needs to be considered. Eastern hemisphere leishmaniasis refers to cases occurring in Old World, whereas Western hemisphere leishmaniasis is found in the Americas (New World). The key differences between Old World and New World leishmaniasis also relate to the causative species, clinical severity, and risk of mucosal involvement. New World cutaneous leishmaniasis shows greater variability in severity. [35] *A detailed travel history to endemic areas is therefore essential. The onset of dactylitis is typically gradual and may be associated with marked skin scarring of the affected digit. Nail involvement is common, with the nail often appearing ischemic and, in some cases, shedding, particularly when nail bed involvement*

(paronychia) is present. The diagnostic work-up should consider patient occupational exposure to farming, along with specific polymerase chain reaction testing. Histopathology may reveal granulomatous histiocytic infiltrates containing Leishmania amastigotes.

Reviewer # 3, Comment 8: **"***More clinical photos are needed especially of psoriatic and sarcoid dactylitis"*

Response: thank you for this helpful suggestion. We have expanded the set of clinical images by including additional cases from our own clinical experience. We have included a collage illustrating representative cases of dactylitis in PsA, gout, chondrocalcinosis and sarcoid dactylitis. These images have been incorporated into the section on the etiologies of dactylitis to provide a clearer comparative overview.

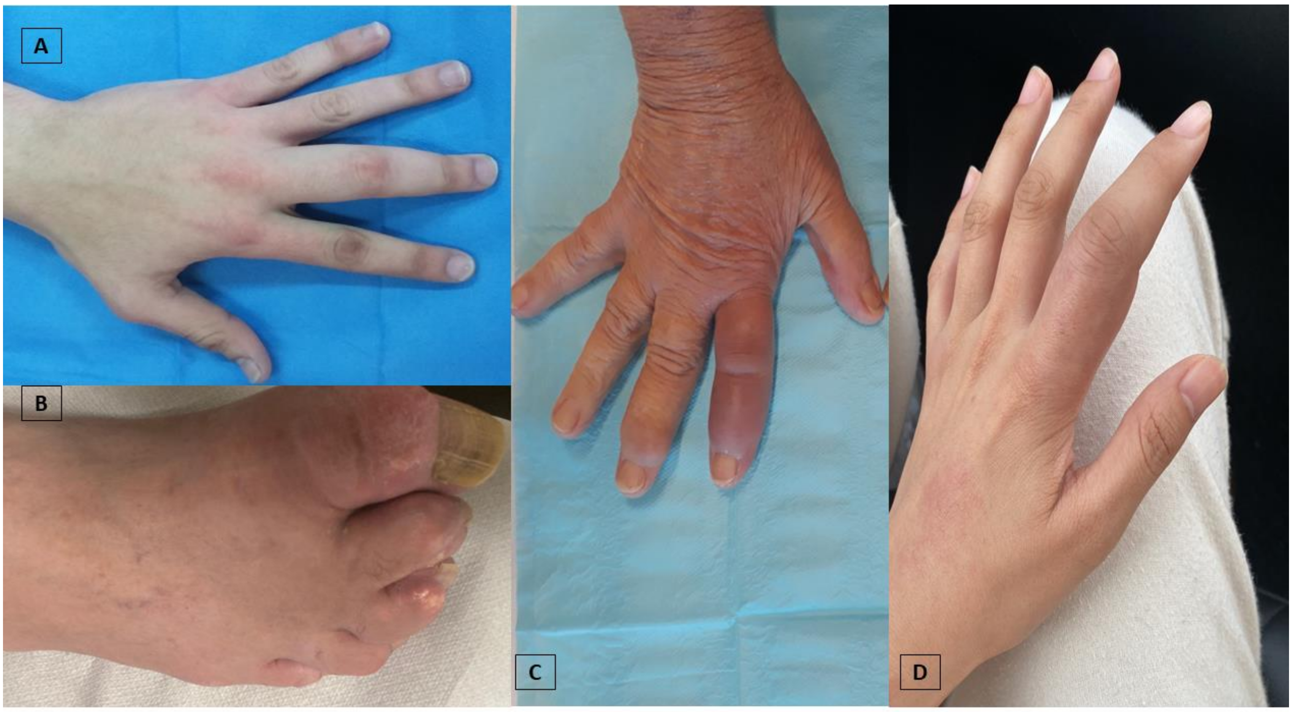

Reviewer # 3, Comment 9: "*The abstract needs to be improved stating its importance in commonly seen conditions such as psoriasis, SpA and sarcoidosis"*

Response: thank you for this valuable comment. We have revised the abstract accordingly, aiming to better highlight the central role of dactylitis in rheumatologic conditions, particularly in psoriatic arthritis, spondyloarthritis, and sarcoidosis**.**

Revised Text:

Dactylitis (from the Greek word dactylos, meaning "digit") is a medical condition commonly known as "sausage digit", referring to the diffuse swelling of a finger or toe.
Dactylitis is most commonly associated with the spectrum of spondyloarthritis, particularly psoriatic arthritis (PsA), and more rarely with other inflammatory conditions such as sarcoidosis and gout. In PsA, dactylitis is associated with increased disease severity and radiographic progression, and its recognition may facilitate early diagnosis and timely treatment. In sarcoid disease, dactylitis is clinically relevant, reflecting granulomatous inflammation and potentially indicating multisystem involvement.

Although most commonly linked to inflammatory disorders, it may also manifest in a variety of other conditions, such as trauma, congenital deformities, infectious diseases, and neoplastic processes, all of which can present with a clinically indistinguishable phenotype.
This review provides a comprehensive, up-to-date overview of dactylitis, beginning with the origin of the term and its earliest historical and scientific references, and concluding with the latest insights into aetiopathogenesis, classification, anatomy, imaging, and therapeutic strategies. Through the integration of clinical knowledge with new developments in imaging, the paper explores how the study of dactylitis can extend beyond psoriatic arthritis.

Reviewer # 3, Comment 10: ***"****Figure 1. There are numbers of the diagram but not explained in the legend"*

Response: thank you for your comment. Figure 1 was originally intended to illustrate the different anatomical zones of the hand according to the surgical classification of Verdan and Kleinert. However, in light of the reviewers' comments and considering the aim of our work—to provide a practical tool for rheumatologists, emphasizing the marked heterogeneity in the etiopathogenesis of dactylitis—we felt that this figure was not essential to the overall message of the manuscript. Therefore, we have decided to remove it.

Reviewer # 3, Comment 11: *"Figure 2. The central slip looks brown rather than red as mentioned in the legend."*

Response: thank you for your comment. We have corrected the figure legend accordingly, changing the color description of the central slip from red to brown.

Reviewer # 3, Comment 12: *"There is no proper Discussion and the concluding statement can be improved and be more informative"*

Response: thank you for this important comment. We have revised the manuscript by adding a dedicated Discussion section to better contextualize the main findings and highlight their clinical implications. In addition, we have improved the concluding statement to provide a clearer and more informative take-home message for the reader.

Revised Text

Discussion

Dactylitis represents a clinical entity of considerable interest for the rheumatologist. In addition to being a marker of common disease such as PsA and other SpAs, it may also represent a non-specific sign of a wide range of disorders, as outlined in this review.

A thorough understanding of the anatomical structures composing the digit, and therefore involved in the development of dactylitis, is essential. In this context, advances in imaging, particularly MSK-US, have a pivotal role in improving diagnostic accuracy. The integration of clinical assessment with the imaging modalities currently available to clinicians allows correct diagnoses in most cases and implementation of more appropriate therapeutic strategies.

In conclusion, dactylitis should be regarded as a multifaceted clinical sign that extends beyond PsA and other forms of PsA and that requires clinicians to consider a number of alternative etiologies.

Ongoing advances in imaging are expected to further enhance our understanding of its pathogenesis and facilitate earlier and more targeted therapeutic interventions

Reviewer # 3, Comment 13: *"The numbering the various sections of the manuscript does not look logical"*

Response: thank you for this comment. We have revised the structure and numbering of the manuscript sections to ensure a more logical and consistent organization throughout the text.
